# Dynamic enhancers control skeletal muscle identity and reprogramming

**DOI:** 10.1371/journal.pbio.3000467

**Published:** 2019-10-07

**Authors:** Krithika Ramachandran, Madhavi D. Senagolage, Meredith A. Sommars, Christopher R. Futtner, Yasuhiro Omura, Amanda L. Allred, Grant D. Barish

**Affiliations:** 1 Department of Medicine, Division of Endocrinology, Metabolism, and Molecular Medicine, Feinberg School of Medicine, Northwestern University, Chicago, Illinois, United States of America; 2 Jesse Brown VA Medical Center, Chicago, Illinois, United States of America; King's College London, UNITED KINGDOM

## Abstract

Skeletal muscles consist of fibers of differing metabolic activities and contractility, which become remodeled in response to chronic exercise, but the epigenomic basis for muscle identity and adaptation remains poorly understood. Here, we used chromatin immunoprecipitation sequencing of dimethylated histone 3 lysine 4 and acetylated histone 3 lysine 27 as well as transposase-accessible chromatin profiling to dissect *cis*-regulatory networks across muscle groups. We demonstrate that in vivo enhancers specify muscles in accordance with myofiber composition, show little resemblance to cultured myotube enhancers, and identify glycolytic and oxidative muscle-specific regulators. Moreover, we find that voluntary wheel running and muscle-specific peroxisome proliferator–activated receptor gamma coactivator-1 alpha (*Pgc1a*) transgenic (mTg) overexpression, which stimulate endurance performance in mice, result in markedly different changes to the epigenome. Exercise predominantly leads to enhancer hypoacetylation, whereas mTg causes hyperacetylation at different sites. Integrative analysis of regulatory regions and gene expression revealed that exercise and mTg are each associated with myocyte enhancer factor (MEF) 2 and estrogen-related receptor (ERR) signaling and transcription of genes promoting oxidative metabolism. However, exercise was additionally associated with regulation by retinoid X receptor (RXR), jun proto-oncogene (JUN), sine oculis homeobox factor (SIX), and other factors. Overall, our work defines the unique enhancer repertoires of skeletal muscles in vivo and reveals that divergent exercise-induced or PGC1α-driven epigenomic programs direct partially convergent transcriptional networks.

## Abstract 2

The epigenomic basis for the identity and adaptation of different muscle groups is poorly understood. This study reveals chromatin landscapes across muscle groups and their dynamic response to either exercise or overexpression of PPAR gamma coactivator 1 alpha.

## Introduction

Skeletal muscle is the most abundant tissue in the body, accounting for approximately 40% of total mass and enabling various movements and postures. In mammals, it is composed of myofibers defined as type I, IIa, IId/x, and IIb based on the expression of myosin heavy chain (Myh) isoforms *Myh7*, *Myh2*, *Myh1*, and *Myh4*, respectively. Oxidative muscles have high levels of *Myh7* and are rich in mitochondria and myoglobin (*Mb*), densely vascularized, and fatigue resistant, thereby supporting endurance. Conversely, glycolytic muscles have high levels of *Myh4* and use glucose and anaerobic respiration, facilitating movements that require rapid energy availability [1]. In spite of overlapping functional and structural elements, each fiber type possesses a unique gene expression profile, including levels of oxidative phosphorylation enzymes and myosin isoforms tailored to metabolic demand and mechanical function. The underlying transcriptional regulators controlling myofiber diversity, however, are incompletely defined.

Skeletal muscles are developmentally bestowed with varying proportions of fiber types, but their metabolic and mechanical properties are highly adaptive to stimulation. Strength training increases fiber size, whereas endurance exercise enhances oxidative metabolism and induces fiber-type transitions [2]. To date, several calcium, metabolite, and hypoxia-responsive cascades have been implicated in exercise-induced adaptation [3], including the Ca^2+^/calmodulin-dependent protein kinase-II, protein kinase C, mitogen-activated protein kinase (MAPK), and AMP-kinase (AMPK) pathways. These in turn control downstream cofactors including histone deacetylases (HDACs) and peroxisome proliferator–activated receptor gamma coactivator-1 (PGC1) and transcription factors such as nuclear factors of activated T cells (NFATs), myocyte enhancer factor (MEF) 2, myogenic regulatory factors (MRFs), peroxisome proliferator–activated receptors (PPARs), estrogen-related receptors (ERRs), sine oculis homeobox factors (SIXs), and hypoxia inducible factors (HIFs). The relative contributions and orchestration of these various mediators in exercise-induced skeletal muscle adaptation are unknown.

Exercise is well documented to mitigate or prevent obesity and cardiometabolic diseases, cancers, and neurocognitive decline [4], which has raised interest in molecules that can be exploited to recapitulate its signaling properties. PGC1α has been among the most studied and, as a transcriptional cofactor, can broadly direct the exercise-induced signals that increase its expression [5]. PGC1α is posttranslationally activated by AMPK as well as sirtuin 1, and it coactivates PPARs, ERRs, nuclear respiratory factors (NRFs), and other interaction partners to drive transcription of oxidative phosphorylation and fiber-specific myofibrillar genes. Accordingly, muscle-specific *Pgc1a* transgenic (mTg) mice have more oxidative myofibers, enhanced mitochondrial activity, and increased endurance performance [6]. The extent to which *Pgc1a* overexpression mimics the signaling effects of exercise at the genomic level is unknown.

Advances in DNA sequencing technology and chromatin-based analysis now enable us to map epigenomic networks on an unprecedented scale and provide opportunities for unbiased discovery of gene regulatory mechanisms. In particular, regulatory region landscapes reflect both cell type–specific developmental origins and dynamic environmental signals directed by lineage determining and signal-dependent transcription factors, respectively [7]. Histone 3 lysine 4 (H3K4) dimethylation (H3K4me2) marks promoters and enhancers and is highly correlated with chromatin accessibility [8–10]. Histone 3 lysine 27 (H3K27) acetylation (H3K27ac) reflects regulatory region activity and has been used in combination with promoter-distal H3K4 monomethylation (H3K4me1) or H3K4me2 to distinguish “poised enhancers” (H3K4me1/2 enriched but lacking H3K27ac signals) from “active enhancers” (H3K4me1/2 and H3K27ac coenriched signals) [10–14]. However, promoter-distal H3K27ac-marked regions without colocalizing H3K4me1 peaks have been associated with similarly high levels of gene expression, suggesting that distal H3K27ac peaks alone may signify active enhancers [11]. Additionally, nucleosome depletion at open regulatory regions renders DNA susceptible to insertion by hyperactive transposase 5 (Tn5) loaded with adapters for high-throughput DNA sequencing, which has been used to identify promoters and enhancers as well as to infer the positions of DNA-bound transcription factors with assay for transposase-accessible chromatin (ATAC) sequencing (ATAC-seq) [15]. Integrative studies of such histone modifications, open chromatin, and RNA expression have dramatically advanced insights into cell- and tissue-specific gene control, differentiation, and disease over the past decade [16–18].

However, high-throughput sequencing–based analyses used to understand the epigenomic regulation of skeletal muscle and exercise have been limited [19,20]. Here, we define mouse skeletal muscle regulatory regions in vivo and determine how exercise or PGC1α modulate their activity using global profiling of H3K4me2, H3K27ac, and ATAC-seq. These epigenomic landscapes revealed significant changes in enhancer activity across muscle groups and conditions, thereby enabling us to comprehensively identify regulators of fiber-type specification and reprogramming.

## Results

### Enhancer repertoires distinguish skeletal muscle groups

**Fig 1:**
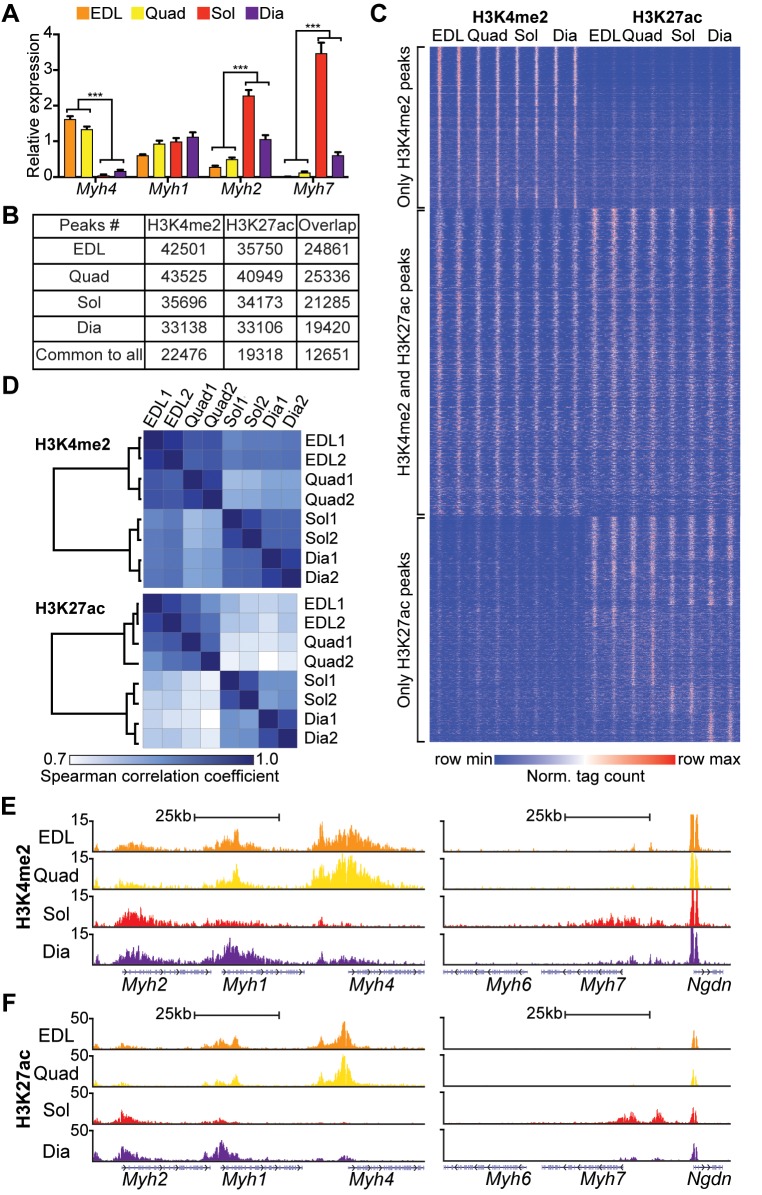
Skeletal muscles exhibit varied *cis*-regulatory repertoires. (A) qPCR expression analysis of *Myh* isoforms measured in different muscle groups (EDL, Quad, Dia, Sol) (*n* = 5/group). Data are represented as means ± SEM. ***p < 0.001. (B) Number of H3K4me2 and H3K27ac peaks identified by ChIP-seq. (C) Heatmap distribution of H3K4me2 and H3K27ac tags within 6 kb of all H3K4me2 and/or H3K27ac peaks identified in EDL, Quad, Sol, and Dia; *n* = 2/group. (D) Similarity matrix for H3K4me2 (top) and H3K27ac (bottom) profiles across muscle groups. Spearman rank correlation coefficient (r_s_) is represented in color. (E,F) Representative UCSC browser tracks of H3K4me2 (E) and H3K27ac (F) ChIP-seq in EDL (orange), Quad (yellow), Sol (red), and Dia (purple) along *Myh* isoform genes. *Myh6* is a cardiac isoform negative control gene, and *Ngdn* is a positive control gene invariant between different muscles. Numerical values for panels A, B, D, E, and F are available in S1 Data, and numerical values for panel C are available in S2 Data, S3 Data and S4 Data. ChIP-seq, chromatin immunoprecipitation sequencing; Dia, diaphragm; EDL, extensor digitorum longus; H3K4me2, histone 3 lysine 4 dimethylation; H3K27ac, histone 3 lysine 27 acetylation; *Myh*, myosin heavy chain; *Ngdn*, neuroguidin; Norm., normalized; qPCR, quantitative PCR; Sol, soleus; Quad, quadriceps femoris; UCSC, University of California, Santa Cruz.

To gain insight into the epigenomic identities for glycolytic and oxidative skeletal muscle, we performed chromatin immunoprecipitation (ChIP) sequencing (ChIP-seq) for H3K4me2 and H3K27ac. Four muscle groups of varied myofiber content, as indicated by their Myh isoforms (**Fig 1A**), were interrogated from biological duplicates of extensor digitorum longus (EDL), quadriceps femoris (Quad), soleus (Sol), and diaphragm (Dia). ChIP-seq for each mark identified over 33,000 high-confidence peaks in each muscle group (**Fig 1B**), with replicates demonstrating highly correlated peaks and normalized tag counts (r_s_ ≥ 0.86) (**S1A and S1B Fig**). Less than 14% of peaks for either mark localized to promoters, and the vast majority were distributed to intragenic and intergenic regions (**S1C Fig**). Among the high-confidence peaks for each muscle group, 20,359 H3K4me2-marked regions and 19,318 H3K27ac-enriched regions were common to all four muscles, with over 62% (12,651 sites) marked by both modifications (**Fig 1B**).

Next, we compared H3K4me2 and H3K27ac data sets between different muscles to gain insight into their epigenomic identities. Global mapping of H3K4me2 and H3K27ac modifications revealed nearly 74,000 aggregated genomic peak locations for either or both marks in skeletal muscle (**Fig 1C**). Of these, between 29% and 33% were H3K4me2-only peaks in each muscle, 41%–47% were H3K4me2 and H3K27ac overlapping peaks, and 20%–29% were H3K27ac-only peaks. Even at sites called only as H3K27ac peaks, H3K4me2 signal was typically present at levels enriched above background, consistent with previous observations that the H3K27ac mark is acquired almost exclusively in the setting of preexisting H3K4 methylation [21]. We performed pairwise comparisons to examine genomic regions enriched for histone modifications. The H3K4me2 mark was highly correlated between EDL and Quad (r_s_ = 0.94) or between Dia and Sol (r_s_ = 0.93), but it was more moderately correlated between Sol and Quad (r_s_ = 0.85) or between Dia and Quad (r_s_ = 0.87) (**S1D Fig**). The H3K27ac mark was likewise strongly associated between EDL and Quad (r_s_ = 0.92), but it was more modestly associated between Dia and Sol (r_s_ = 0.86) and even less consistent between either Sol and Quad or Dia and Quad (r_s_ = 0.76, for both) **(S1E Fig)**. To extend these findings, we performed hierarchical clustering using the aggregate of peaks identified in the four different muscle groups for each mark. H3K4me2 marks clustered closely between Dia and Sol but were more distinct between either of these muscles and the glycolytic EDL or Quad groups (**Fig 1D,** top). H3K27ac enrichment was highly correlated between Dia and Sol or between EDL and Quad (**Fig 1D,** bottom). As seen in pairwise analysis (**S1D and S1E Fig**), four-group hierarchical clustering further demonstrated a greater variation in genome-wide H3K27ac than H3K4me2 signals across different muscles. In EDL and Quad, visualization of the *Myh2/1/4* locus revealed enrichment of H3K4me2 and H3K27ac along *Myh4* (**Fig 1E and 1F**), consistent with their predominantly glycolytic fiber compositions (**Fig 1A**). By contrast, in Sol and Dia, these marks were attenuated at *Myh4* but increased at the oxidative fiber components *Myh2* and *Myh7* (**Fig 1E and 1F**).

**Fig 2:**
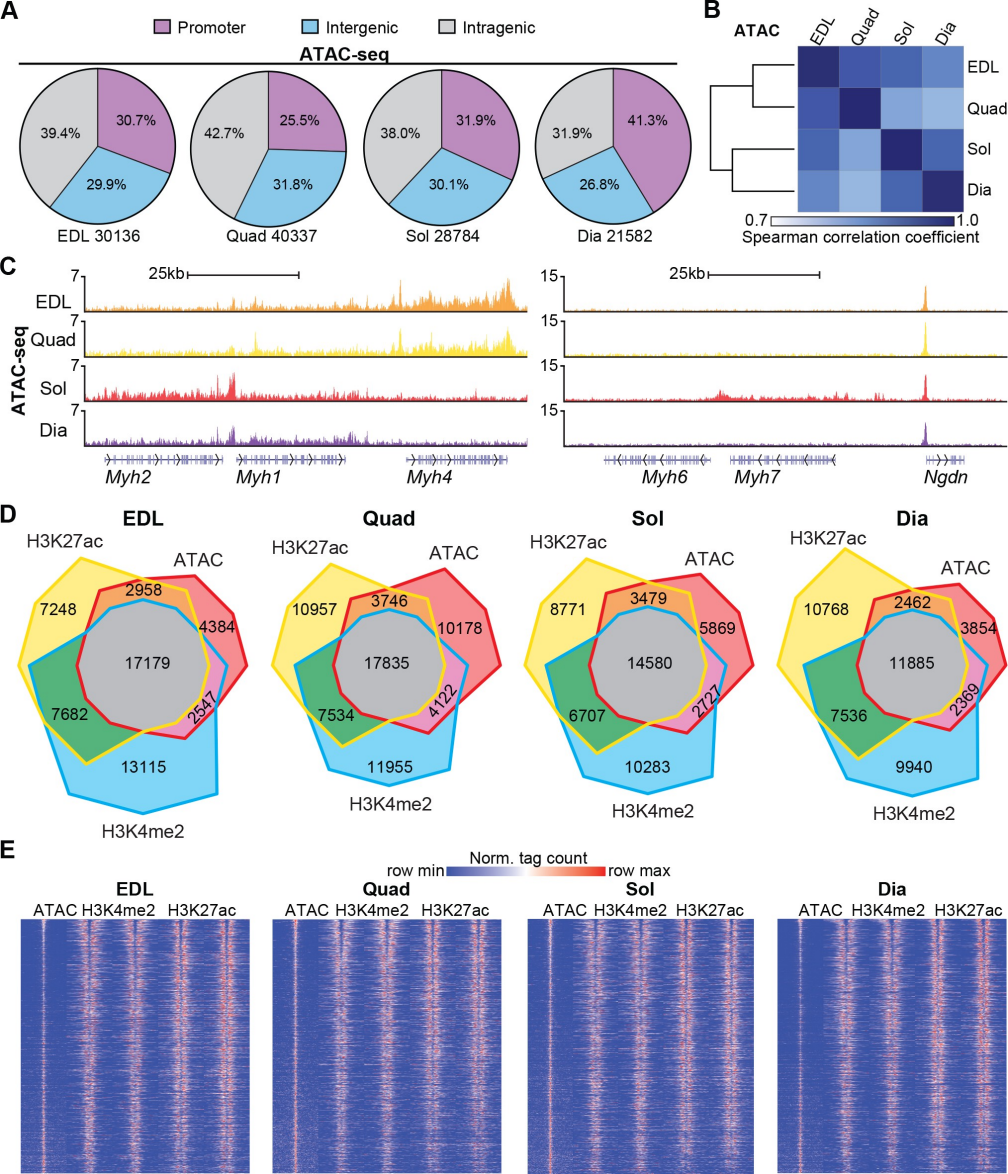
Patterns of open chromatin differ across muscle groups. (A) Genomic distributions of ATAC-seq peaks across EDL, Quad, Sol, and Dia in promoter, intragenic, and intergenic regions. (B) Similarity matrix for ATAC-seq profiles across muscle groups. Spearman rank correlation coefficient (r_s_) is represented in color. (C) Representative UCSC browser tracks of ATAC-seq in EDL (orange), Quad (yellow), Sol (red), and Dia (purple) along *Myh* isoform genes. *Myh6* is a cardiac isoform negative control gene, and *Ngdn* is a positive control gene invariant between different muscles. (D) Venn diagrams showing overlapping and unique regulatory regions (H3K4me2, H3K27ac, and ATAC-seq peaks) in EDL, Quad, Sol, and Dia. (E) Heatmap distribution of ATAC-seq, H3K4me2, and H3K27ac tags within 4 kb of all ATAC-seq peaks identified in EDL, Quad, Sol, and Dia; *n* = 1–2/group. Numerical values for panels A, B, C, and D are available in S1 Data, and numerical values for panel E are available in S5 Data, S6 Data, S7 Data, and S8 Data. ATAC-seq, assay for transposase-accessible chromatin sequencing; Dia, diaphragm; EDL, extensor digitorum longus; H3K4me2, histone 3 lysine 4 dimethylation; H3K27ac, histone 3 lysine 27 acetylation; *Myh*, myosin heavy chain; *Ngdn*, neuroguidin; Norm., normalized; Quad, quadriceps femoris; Sol, soleus; UCSC, University of California, Santa Cruz.

We performed ATAC-seq to map open regulatory regions and identified over 20,000 peaks for each muscle, 25%–41% of which mapped to gene promoters (**Fig 2A**). Hierarchical clustering showed that open chromatin was highly correlated among all groups (Spearman correlation coefficients > 0.75), although EDL and Quad or Sol and Dia were most similar (**Fig 2B**) and mirrored the relationships obtained for ChIP-seq enrichments of H3K4me2 or H3K27ac (**Fig 1D**). Likewise, visualization of ATAC-seq peaks along *Myh* gene loci detected openness in EDL and Quad along *Myh4* or in Sol and Dia near *Myh2* and, to a modest extent, near *Myh7* (**Fig 2C**). Integration with our ChIP-seq data sets for each muscle group revealed that >75% of ATAC-seq peaks colocalized with H3K4me2- and/or H3K27ac-enriched regions (**Fig 2D**), which surrounded these sites of nucleosome depletion (**Fig 2E**).

To assess the regulatory impact of muscle group–specific chromatin, we examined the relationship between H3K27ac enrichment or transposase accessibility and corresponding gene transcription using previously published muscle mRNA sequencing (mRNA-seq) data sets [22]. Hierarchical clustering of EDL, Quad, Sol, and Dia expression patterns were analogous to those derived from our ChIP-seq and ATAC-seq data sets, with EDL and Quad clustered separately from Sol and Dia (**S2A Fig**). Interrogation of genes expressed at significantly higher levels in Quad compared with Sol revealed corresponding changes in H3K27ac and ATAC-seq signals within a 50-kb window surrounding transcription start sites (TSSs) (**S2B and S2C Fig**, left). The opposite was observed near genes expressed at significantly higher levels in Sol (**S2B and S2C Fig**, right). In sum, these analyses provided strong evidence that H3K4me2, open chromatin, and, to an even larger extent, H3K27ac landscapes accurately distinguish muscle groups in accordance with their transcriptional and myofiber profiles.

### Skeletal muscle groups exhibit diverse super-enhancers

**Fig 3:**
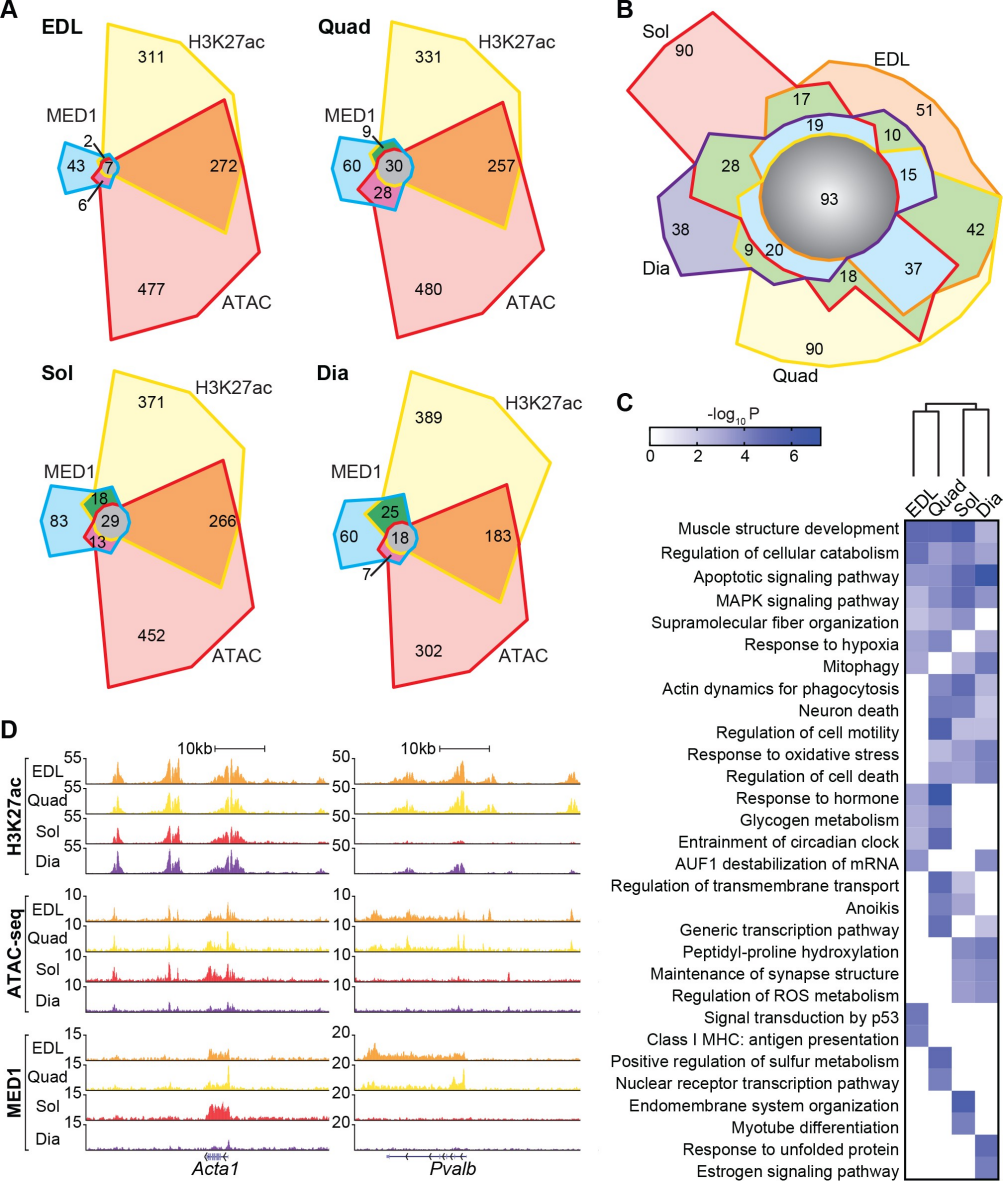
SEs are distinct among muscle groups. (A) Venn diagrams showing overlapping and unique SEs identified using three different methods (H3K27ac, Med1, and ATAC-seq) in EDL, Quad, Dia and Sol. (B) Overlap between the high-confidence SEs across the four muscle groups. High-confidence SEs were defined as those identified by at least two out of the three methods. (C) Heatmap of top ontologies for genes annotated to the high-confidence SEs in EDL, Quad, Sol, and Dia, with *p*-values represented in color. (D) Representative UCSC browser tracks of H3K27ac ChIP-seq (top), ATAC-seq (middle), and MED1 ChIP-seq (bottom) in EDL (orange), Quad (yellow), Sol (red), and Dia (purple) at a SE along *Acta1* common to all muscles (left) and a glycolytic muscle-specific SE along *Pvalb* (right). Numerical values for all panels are available in S1 Data. *Acta1*, actin alpha-1; ATAC, assay for transposase-accessible chromatin sequencing; ATAC-seq, ATAC sequencing; AUF1, AU rich element RNA binding protein 1; ChIP-seq, chromatin immunoprecipitation sequencing; Dia, diaphragm; EDL, extensor digitorum longus; H3K27ac, histone 3 lysine 27 acetylation; MAPK, mitogen-activated protein kinase; Med1, mediator complex subunit 1; MHC, myosin heavy chain; *Pvalb*, parvalbumin; Quad, quadriceps femoris; ROS, reactive oxygen species; SE, super-enhancer; Sol, soleus; UCSC, University of California, Santa Cruz.

Next, we further leveraged our H3K27ac and ATAC-seq data sets and performed ChIP-seq in each muscle for mediator complex subunit 1 (MED1) to identify super-enhancers (SEs) (**Fig 3A**), specialized enhancers spanning particularly long DNA domains and exhibiting heavy H3K27ac and H3K4me1 modification, open chromatin, and occupancy by cell type–specific master transcription factors and mediator [23]. Analysis of H3K27ac or ATAC-seq identified several hundreds of SEs in muscles, whereas MED1 data yielded more limited numbers in each group, consistent with findings in other cells or tissues [23,24]. Complete sets of SEs for each muscle were annotated to their nearest gene, and we defined high-confidence SEs found by at least two out of our three identifiers **(S9 Data**). Less than 40% of high-confidence muscle SEs were common to all muscle groups (**Fig 3B**), and these were particularly enriched at genes involved in muscle structure, cellular catabolism, apoptosis, and MAPK signaling (**Fig 3C**). As many as one-quarter of high-confidence muscle SEs were group specific, and gene ontologies for the SEs of EDL, Quad, Sol, and Dia reflected features of their distinct physiology including enrichment for glycogen metabolism in EDL and quadriceps or response to oxidative stress and reactive oxygen species metabolism for Sol and Dia. Direct visualization of H3K27ac and MED1 ChIP-seq and ATAC-seq tracks confirmed the existence of common and group-specific SEs along genes such as actin alpha-1 (*Acta1*) and parvalbumin (*Pvalb*), respectively (**Fig 3D**). Thus, a majority of regulatory regions are shared, but up to one-quarter of SEs differ between skeletal muscles.

### In vitro and in vivo muscle regulatory regions are highly divergent

**Fig 4:**
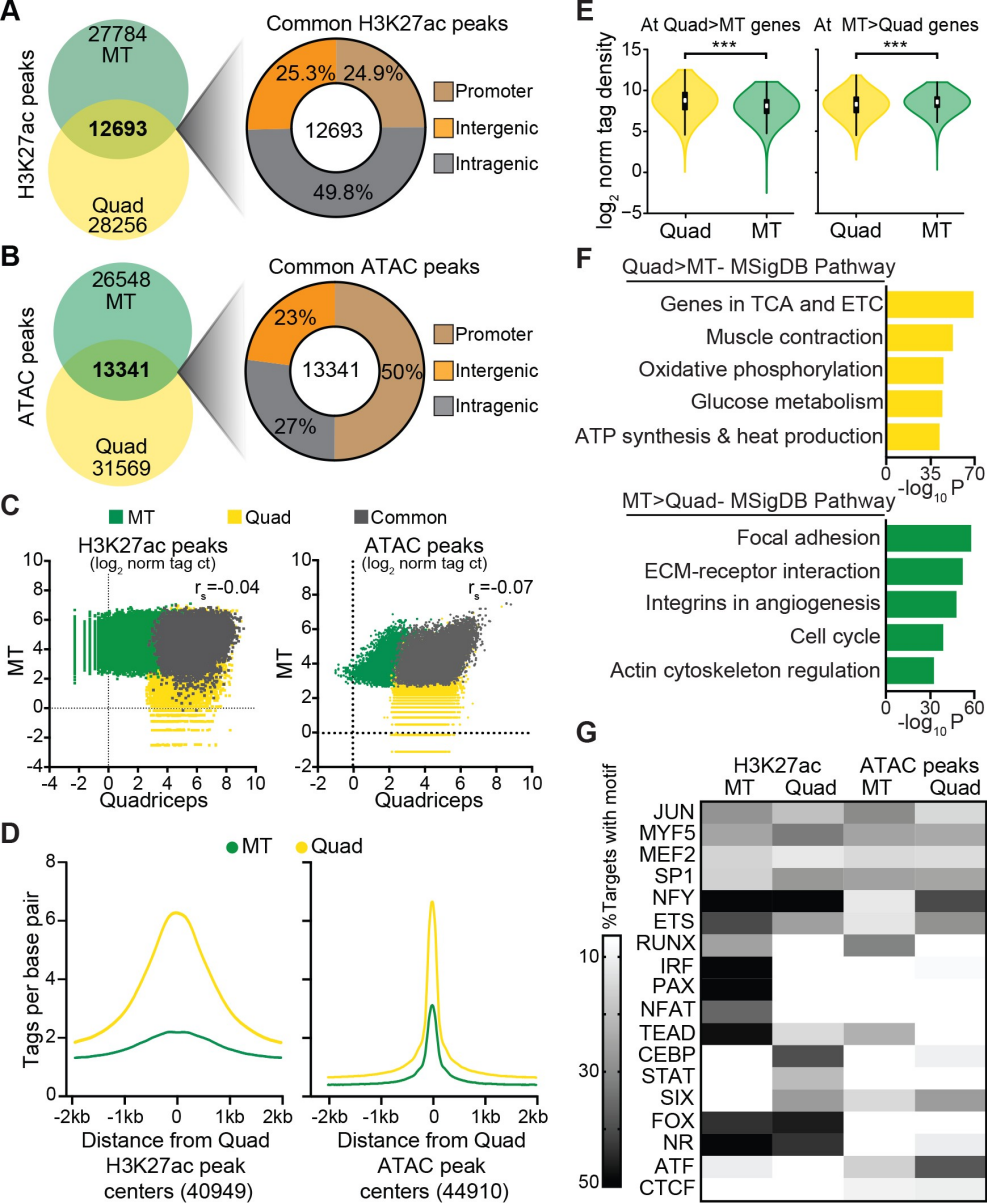
Enhancer repertoires are uncorrelated between MTs in vitro and muscle tissue in vivo. (A,B) Venn diagram showing overlapping and unique (A) H3K27ac and (B) ATAC peaks in C2C12 MT and Quad (left). Genomic distributions of the peaks common to MT and Quad in promoter, intergenic, and intragenic regions are shown (right). (C) Scatterplots of normalized H3K27ac (left) and ATAC (right) tag counts at peaks identified in MT, Quad, or both, respectively. The correlation coefficient (r_s_) is listed. (D) Histogram of H3K27ac (left) and ATAC (right) tags in MT and Quad centered on all peaks identified in Quad. (E) H3K27ac tags within 50 kb around the TSS for genes differentially expressed in Quad > MT (left) or MT > Quad (right); *n* = 2/group, ***p < 0.001. Violin plots display median (white square), interquartile range (black rectangle), 95% confidence interval (black line), and frequency (colored density plot). (F) Top ontologies for H3K27ac regions that annotate near genes expressed in Quad > MT (top) and MT > Quad (bottom). (G) Heatmap showing percentage of H3K27ac and ATAC peaks containing the top transcription factor motifs in MT or Quad. Numerical values for all panels are available in S1 Data. ATAC, assay for transposase-accessible chromatin; ETC, electron transport chain; ECM, extracellular matrix; ETS, E-twenty-six family of transcription factors; H3K27ac, histone 3 lysine 27 acetylation; Jun, jun proto-oncogene; MSigDB, Molecular Signatures database; MEF2, myocyte enhancer factor 2; MYF5, myogenic factor 5; NFY, nuclear transcription factor Y; MT, myotube; norm tag ct, normalized tag count; PAX, paired box; Quad, quadriceps femoris; RUNX, runt-related transcription factor; SP1, specificity protein 1; TCA, citric acid cycle; TEAD, TEA domain transcription factor 1; TSS, transcription start site.

Previous studies have explored the epigenomic transition of myoblasts to myotubes (MTs) [25,26]. However, the extent to which muscle cells differentiated in vitro reflect genomic architecture in vivo has not been established. To address this, we compared published H3K27ac ChIP-seq data sets from differentiated C2C12 MTs to our data derived from muscle tissue. We found that fewer than one-third of H3K27ac-marked regions were shared between MT and Quad (**Fig 4A**), and peak overlaps were disproportionately detected at promoter regions, which represented 25% of overlapping peaks. To further examine the regulatory landscapes of muscle in vitro and in vivo, we compared open regulatory regions in Quad to published ATAC-seq data in MT. Like our ChIP-seq findings, fewer than one-third of ATAC-seq peaks were commonly identified in MT and Quad, and 50% of these common peaks annotated to promoter regions (**Fig 4B**). Analysis of tag densities at all peaks identified in MT or Quad revealed no correlation in H3K27ac or ATAC-seq signal (**Fig 4C**). Accordingly, at Quad-specific H3K27ac-marked regions, there was virtually no enrichment for this modification in vitro (**Fig 4D**, left). Similarly, at Quad-specific ATAC-seq sites, peak strength was greatly attenuated in MT compared with muscle tissue (**Fig 4D**, right). These observations indicated a high degree of divergence between in vivo and in vitro muscle regulatory landscapes.

Next, we extended our analysis to understand the impact of Quad and MT regulatory regions on gene activity. Interrogation of genes expressed at significantly higher levels in Quad compared with MT revealed corresponding changes in H3K27ac within a 50-kb window surrounding TSSs (**Fig 4E**, left). The converse was observed near genes expressed at significantly higher levels in MTs, although the relative enrichment in MT H3K27ac signal was more modest (**Fig 4E**, right). Gene ontology analysis for H3K27ac peaks associated with genes more highly expressed in Quad versus MT or vice versa identified metabolic and mitochondrial or extracellular matrix and cell cycle categories, respectively (**Fig 4F**). We also compared motifs within H3K27ac or ATAC-seq peaks and found similar representations of jun proto-oncogene (JUN), myogenic factor 5 (MYF5), MEF2, specificity protein 1 (SP1), nuclear transcription factor Y (NFY), and E-twenty-six family transcription factor (ETS) elements in MT and Quad, whereas factors critical for progenitor functions including runt-related transcription factor (RUNX), paired box (PAX), and TEA domain transcription factor 1 (TEAD) were more concentrated in MT (**Fig 4G**). These findings together suggested that although myotubular differentiation involves many common transcription factors in vitro and in vivo, muscle transcriptional control is highly distinctive in tissue.

### Unique regulatory regions reflect muscle group–specific identity

**Fig 5:**
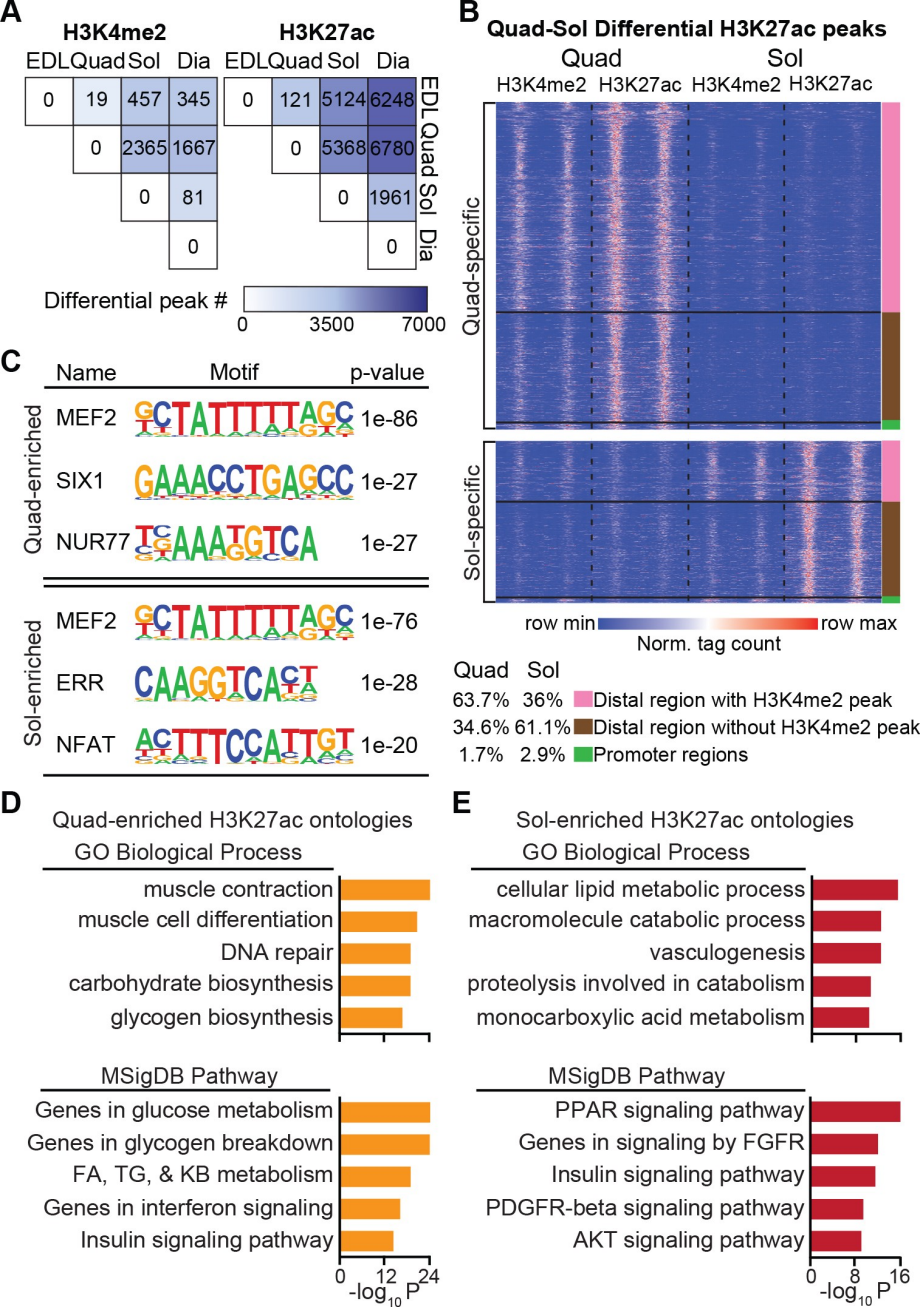
Enhancer regions reflect muscle group–specific function. (A) Heatmap of pairwise comparisons showing the number of differential H3K4me2 (left) and H3K27ac (right) peaks in EDL, Quad, Dia, and Sol. Differential peak number is represented by color. (B) Heatmap distribution of H3K4me2 and H3K27ac tags within 6 kb of Quad-specific (top) and Sol-specific (bottom) H3K27ac peaks in Quad and Sol; *n* = 2/group. Color bars on the right correspond to promoter regions, promoter-distal regions with H3K4me2 peaks, or promoter-distal regions without H3K4me2 peaks. (C) Top three motifs enriched within H3K27ac peaks in Quad compared with Sol (top) and Sol compared with Quad (bottom). (D, E) Top five categories of genes annotated to H3K27ac peaks specific to Quad (D) or Sol (E), respectively. Numerical values for panels A, C, D, and E are available in S1 Data, and numerical values for panel B are available in S10 Data. AKT, protein kinase B; Dia, diaphragm; EDL, extensor digitorum longus; FA, fatty acid; FGFR, fibroblast growth factor receptor; GO, gene ontology; H3K4me2, histone 3 lysine 4 dimethylation; H3K27ac, histone 3 lysine 27 acetylation; KB, ketone body; MSigDB, Molecular Signatures database; MEF, myocyte enhancer factor; NFAT, nuclear factor of activated T cells; Norm., normalized; NUR77, nuclear hormone receptor 77; PDGFR, platelet-derived growth factor receptor; PPAR, peroxisome proliferator–activated receptor; Quad, quadriceps femoris; SIX, sine oculis homeobox factor; Sol, soleus; TG, triacylglycerol.

We hypothesized that histone marks could be leveraged to discover the basis for muscle group–specific transcriptional programming in vivo. To test this, we first sought to identify regions that were uniquely or more heavily marked in certain muscles compared with others. We defined group-specific H3K4me2- and H3K27ac-enriched regions using differential peak calling in pairwise comparisons between EDL, Quad, Sol, and Dia. For H3K4me2, few regions of altered signal were detected between muscle groups (**Fig 5A,** left). Distinctions in H3K4me2 landscapes were most apparent between Quad and Sol or Dia, with 2,365 or 1,667 altered H3K4me2 regions, respectively, again indicating that metabolically distinct muscle groups are epigenomically distinguishable. Notably, however, enrichment for H3K27ac was considerably more dynamic, resulting in 2–24 times as many differentially marked regions in pairwise comparisons (**Fig 5A,** right). The greatest number of differential peaks was detected in EDL or Quad versus Sol or Dia, with over 5,000 differentially acetylated regions found in each of these comparisons. Direct visualization of H3K4me2 and H3K27ac enrichment along regions differentially acetylated between Quad and Sol reflected the highly dynamic H3K27ac signals between muscle groups (**Fig 5B**). Over 97% of differentially acetylated sites localized outside of promoter regions. Virtually all colocalized with increased H3K4me2 signals, although fewer than two-thirds of differentially acetylated sites were formally called as H3K4me2 peaks. Thus, altered *cis*-regulatory region activity as reflected in H3K27ac, more so than repertoire indicated by H3K4me2, separates muscle group epigenomes.

We focused on H3K27ac regions to distinguish muscle groups using transcription factor motif discovery and gene annotation. Global H3K27ac data sets for EDL, Quad, Sol, and Dia revealed similar motif profiles (**S1F Fig**). However, analysis of differentially H3K27ac regions between predominantly glycolytic and oxidative muscles identified some unique motif enrichments. For example, we found that hyperacetylated regions in Quad when compared with H3K27ac peaks in Sol were enriched in consensus sequences for the muscle developmental regulators MEF2 [27] and SIX1 [28], as well as nuclear hormone receptor 77 (NUR77) (**Fig 5C,** top). Levels of each of these mRNAs were also enriched in Quad compared with Sol (**S3A Fig)**. Conversely, hyperacetylated regions in Sol compared with Quad were enriched in motifs for MEF2 and the oxidative metabolic regulators ERR and NFAT (**Fig 5C,** bottom) [29,30]. Likewise, expression levels of *Errb* and *Errg* were increased, but *Nfatc1* was somewhat reduced in Sol (**S3A Fig**). To test the veracity of our motif discovery, we performed ChIP for NFATc1 and SIX1 in Quad and Sol. ChIP-seq revealed 50% increased NFATc1 occupancy in Sol compared with Quad at motif-predicted sites within Sol-specific H3K27ac peaks (**S3B Fig**), including the mitochondrial enzyme 3-hydroxybutyrate dehydrogenase 1 (*Bdh1*) locus (**S3C Fig**). ChIP quantitative PCR (qPCR) demonstrated SIX1 occupancy along Quad-specific hyperacetylated peaks (**S3D Fig**). Interestingly, SIX1 enrichment was generally lower in Quad than in Sol at these binding sites, consistent with reported roles for SIXs as repressors [31,32]. Additionally, we annotated Quad and Sol differentially acetylated peaks to nearby genes. Quad-enriched regions annotated to genes involved in muscle contraction and differentiation and carbohydrate and glucose metabolism (**Fig 5D**), mirroring its predominantly glycolytic profile. By contrast, differential H3K27ac peaks from Sol localized to genes involved in lipid metabolism, vasculogenesis, and PPAR signaling (**Fig 5E**), reflecting an oxidative program. Together, these findings highlighted the predictive potential of epigenomic profiling to identify muscle group–specific *trans*-acting factors and metabolic state.

### Exercise and PGC1α distinctively remodel muscle chromatin

**Fig 6:**
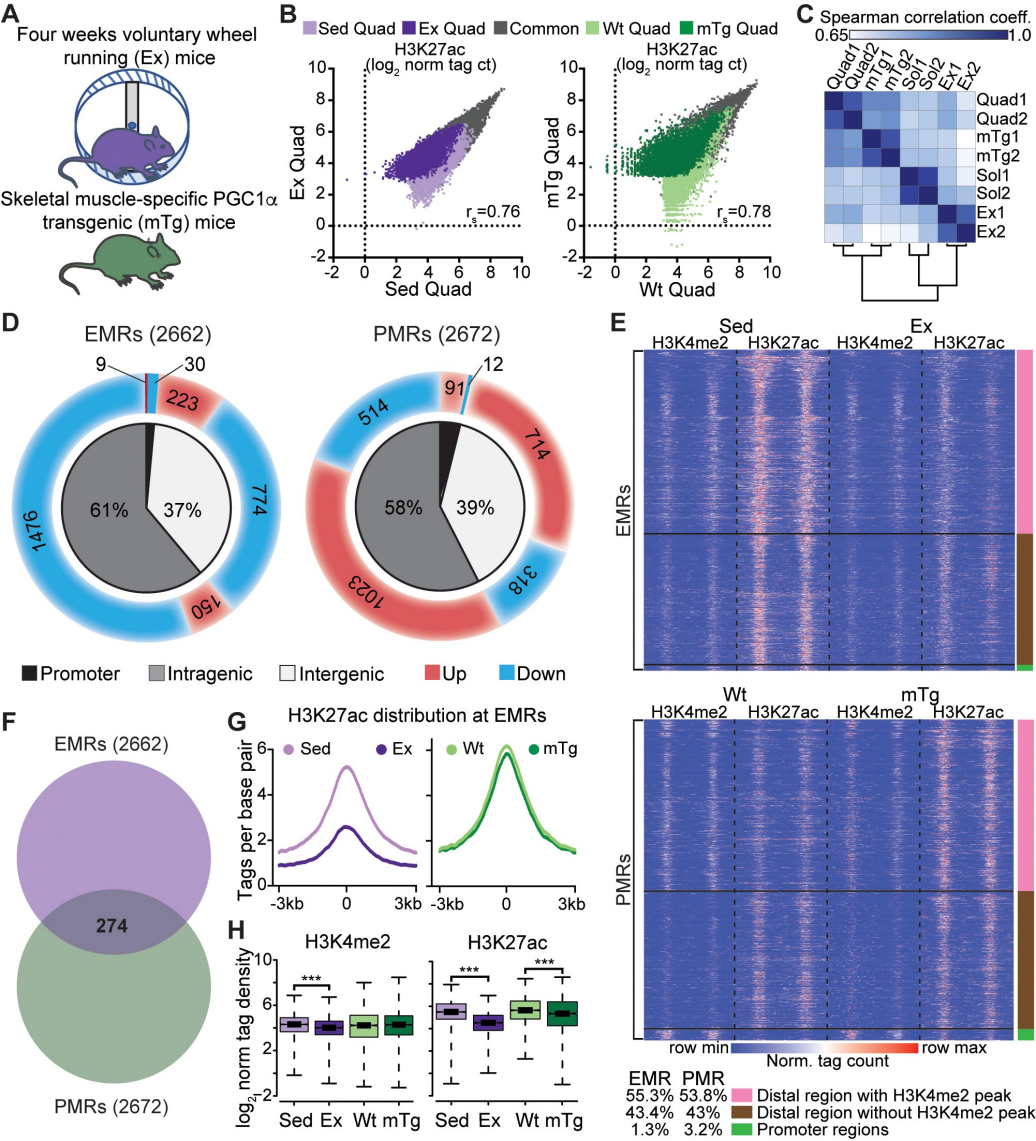
Exercise and PGC1α modify the epigenome at different regions. (A) Experimental scheme of voluntary wheel running (Ex) and skeletal muscle–specific *Pgc1a* overexpression (mTg). Ex mice were placed in cages with wheels for 4 weeks. (B) Scatterplots of normalized H3K27ac tag counts at regions marked by H3K27ac in Sed versus Ex Quad (left) and Wt versus mTg Quad (right). Correlation coefficient (r_s_) was calculated for each scatterplot; *n* = 2/group. (C) Similarity matrix with Spearman correlations for H3K27ac profiles in Quad and Sol, Ex and mTg Quad with correlation coefficient (r_s_) in color. (D) Pie charts with locations and H3K27ac signal changes at EMRs and mTg-modified regions (PMRs). Inner charts show peak distributions in promoter, intragenic, and intergenic regions. Outer charts indicate the direction of the acetylation change, with red for hyperacetylation and blue for hypoacetylation. (E) Heatmap distribution of H3K4me2 and H3K27ac tags within 6 kb of EMR (top) and PMR (bottom) peaks in Sed versus Ex Quads and Wt versus mTg Quads, respectively; *n* = 2/group. Color bars on the right correspond to promoter regions, promoter-distal regions with H3K4me2 peaks, or promoter-distal regions without H3K4me2 peaks. (F) Venn diagram with overlap between EMRs and PMRs. (G) Histograms showing distribution of H3K27ac tags within 6 kb of EMR peak centers in Ex and mTg mice and controls; *n* = 2/group. (H) Quantification of H3K4me2 (left) and H3K27ac (right) tag densities at EMR H3K27ac peaks in Sed, Ex, Wt, and mTg Quads. *n* = 2/group, ***p < 0.001. Numerical values for panels B, C, D, F, G, and H are available in S1 Data, and numerical values for panel E are available in S11 Data. EMR, exercise-modified region; Ex, exercised; H3K4me2, histone 3 lysine 4 dimethylation; H3K27ac, histone 3 lysine 27 acetylation; mTg, muscle-specific *Pgc1a* transgenic; norm tag ct, normalized tag count; *Pgc1a*, peroxisome proliferator–activated receptor gamma, coactivator 1 alpha; PMR, PGC1α-modified region; Quad, quadriceps femoris; Sed, sedentary; Sol, soleus; Wt, wild-type.

Having established epigenomic landscapes for glycolytic and oxidative muscles bestowed by development, we next sought to use histone regulatory marks to gain insight into the transcriptional basis for exercise-induced skeletal muscle adaptation and oxidative reprogramming. To do so, we profiled wild-type C57BL/6J mice subjected to voluntary wheel running for 4 weeks (exercised [Ex]) (**Fig 6A**), a highly consistent exercise program (**S4A Fig**) known to promote oxidative transitions in myofibers [33]. Additionally, we analyzed mTg mice that constitutively express *Pgc1a* under control of the muscle creatine kinase promoter, endowing them with increased oxidative fiber content and endurance in glycolytic muscles (**Fig 6A**). In these animals, transgenic expression raises *Pgc1a* in glycolytic muscles to levels comparable to its high endogenous expression in Sol [6].

Next, we interrogated the chromatin regulatory landscape of Ex and mTg mice in a manner similar to our previous analysis. ChIP-seq in the Quad of Ex or mTg mice revealed that H3K4me2 marks were highly correlated (r_s_ = 0.95 or 0.89, respectively) compared with Quad from untrained wild-type controls (**S4B Fig**). Accordingly, no differentially dimethylated regions were identified in Ex Quad, and only 32 differentially H3K4me2-marked regions were found in mTg Quad compared with controls (**S4C Fig**). However, H3K27ac was far more dynamic (**Fig 6B**). Additionally, we performed an unsupervised clustering of all H3K27ac-marked regions identified in Quad from all experimental conditions (untrained, Ex, and mTg) and Sol of untrained mice (**Fig 6C**). This analysis revealed that untrained and mTg Quad share a high degree of similarity, whereas Ex Quad exhibits the most disparate H3K27ac landscape. These findings led us to conclude that overexpression of *Pgc1a* reprograms the H3K27ac landscape to an intermediate profile between untrained Quad and untrained Sol, whereas exercise shifts Quad regulatory region activity to more closely resemble an untrained Sol than an untrained Quad.

To understand the epigenomic impact of exercise and the degree to which it is mimicked by *Pgc1a* transgenesis, we focused on differentially acetylated H3K27 peaks and identified over 2,600 regions in Ex or mTg muscle compared with controls. More than 96% of these exercise-modified regions (EMRs) or PGC1α-modified regions (PMRs) localized outside of gene promoters (**Fig 6D**). More than half of EMRs and PMRs colocalized with H3K4me2 peaks, and the remainder generally exhibited lower but apparent H3K4me2 signals suggestive of active enhancers (**Fig 6E**). Over 85% (2,280/2,662) of EMRs were hypoacetylated in Ex Quad (**Fig 6E,** top), whereas more than 68% (1,828/2,672) of PMRs were hyperacetylated in mTg Quad (**Fig 6E,** bottom). Remarkably, only 10% of differentially acetylated genomic regions (274 peaks) were cotargeted by exercise and *Pgc1a* overexpression (**Fig 6F**). These findings suggested that exercise targets enhancers that appear PGC1α independent, and it more often suppresses than increases their activity.

To further examine differentially acetylated sites, we quantified H3K27ac and H3K4me2 marks centered on EMRs and PMRs in Ex and mTg mice as well as their respective controls. At EMRs, Ex mice generally exhibited H3K27ac hypoacetylation (**Fig 6G**). Quantitative analysis of H3K4me2 and H3K27ac marks within 1 kb of the EMR centers confirmed significant overall exercise-induced reductions of each modification, with 18% loss in H3K4me2 and 50% loss in H3K27ac (**Fig 6H**). Additionally, to test the durability of H3K27ac changes at EMRs, we subjected mice to 4 weeks of voluntary wheel running but removed wheels 72 hours prior to tissue harvest for H3K27ac ChIP-seq. Significant hypoacetylation (11% reduced) persisted at EMRs in these detraining mice (**S4D Fig**), although the difference was more moderate than in continuously trained animals. In mTg mice, we also observed a modest yet significant (7%) overall reduction in H3K27ac signal at EMRs but no change in H3K4me2 (**Fig 6H**). At PMRs, H3K4me2 marks were slightly reduced in both Ex and mTg mice, whereas H3K27ac marks were accentuated by 60% in mTg mice but diminished by 25% in Ex mice (**S4E and S4F Fig**). Notably, mTg muscles had enriched levels of *F4/80* and *Cd11b*, as well as *Cd31* and vascular endothelial growth factor A (*Vegfa*), in line with their reportedly increased tissue-resident macrophage content and vascularity (**S4G Fig**) [34,35]. However, qPCR of markers for immune cells, vascular cells, satellite cells, myoblasts (myogenic differentiation 1 [*MyoD*]), and MTs (myogenin [*MyoG*]) indicated no changes in the cellular composition of Ex muscles despite higher *Mb* levels from chronic training (**S4G Fig**). These findings suggested that the epigenome of Ex muscle reflects its native cell population, whereas altered cellularity could contribute to signals in mTg tissue. Overall, these findings indicated that exercise and PGC1α differentially target a subgroup of skeletal muscle *cis*-regulatory domains and their activities.

**Fig 7:**
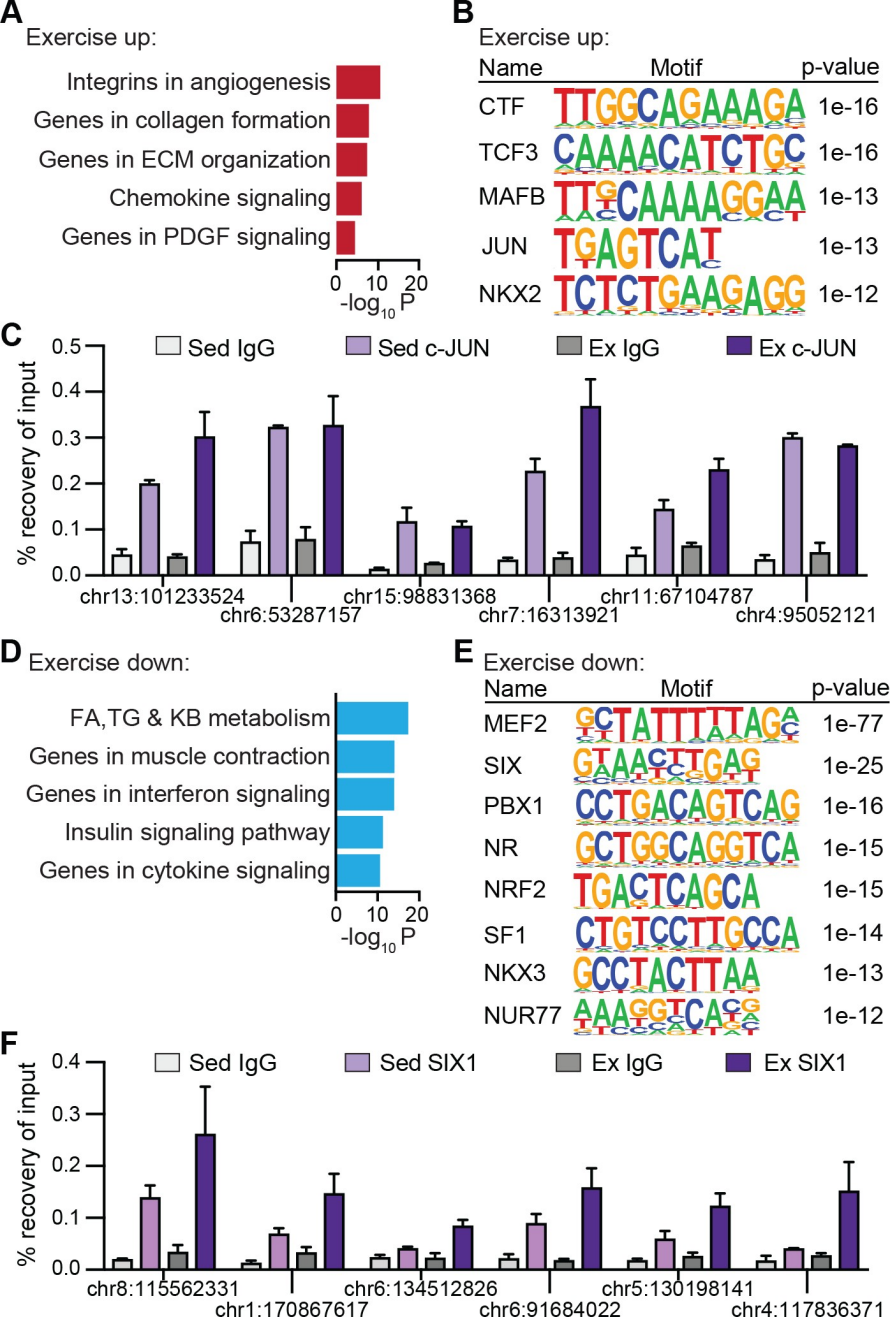
Motif predictions and validations at EMRs. (A) Top ontologies for genes annotated near hyperacetylated H3K27ac regions in Ex Quad. (B) Motifs enriched at hyperacetylated EMRs. (C) ChIP qPCR validation of c-JUN binding in Sed and Ex Quad. Enrichment is plotted as percent of input using technical duplicate control IgG and c-JUN ChIPs. (D) Top ontologies for genes annotated near hypoacetylated H3K27ac regions in Ex Quad. (E) Motifs enriched at hypoacetylated EMRs. (F) ChIP qPCR validation of SIX1 binding in Sed and Ex Quad. Enrichment is plotted as percent of input using technical duplicate control IgG and SIX1 ChIPs. Numerical values for all panels are available in S1 Data. CTF, CCAAT box-binding transcription factor; ChIP, chromatin immunoprecipitation; chr, chromosome; EMR, exercise-modified region; Ex, exercised; ECM, extracellular matrix; FA, fatty acid; H3K27ac, histone 3 lysine 27 acetylation; IgG, immunoglobulin G; Jun, jun proto-oncogene; KB, ketone body; MAFB, MAF bZIP transcription factor B; NKX2, NK2 homeobox; NUR77, nuclear hormone receptor 77; NR, nuclear receptor; PBX1, pre-B-cell leukemia homeobox 1; PDGF, platelet-derived growth factor; qPCR, quantitative PCR; Quad, quadriceps femoris; Sed, sedentary; SIX, sine oculis homeobox factor; SF1 steroidogenic factor 1; TCF3, transcription factor 3; TG, triacylglycerol.

We next performed DNA motif analyses at hyper- and hypoacetylated peak sets for both EMRs and PMRs and annotated these to nearby genes. Hyperacetylated EMRs localized to genes involved angiogenesis, matrix remodeling, and chemokine and platelet-derived growth factor (PDGF) signaling (**Fig 7A**) and were enriched for basic leucine zipper domain factors (bZIP) (JUN, MAF bZIP transcription factor B [MAFB]), CCAAT box-binding transcription factor (CTF), transcription factor 3 (TCF3), and NK2 homeobox (NKX2) binding sites (**Fig 7B**). Consistent with motif predictions, ChIP qPCR analysis confirmed occupancy of c-JUN at a subset of hyperacetylated EMRs (**Fig 7C**). In contrast, hyperacetylated PMRs mapped to genes controlling lipid metabolism (**S4H Fig,** top) and were overrepresented in MEF2 and ERR motifs (**S4I Fig,** left). Hypoacetylated EMRs localized to lipid metabolic and muscle contraction genes as well as interferon, insulin, and cytokine signaling components (**Fig 7D**) and were enriched in MEF2, SIX, pre-B-cell leukemia homeobox1 (PBX1), nuclear receptor (NR), and NRF motifs (**Fig 7E**). In line with motif predictions, SIX1 binding was detected at a subset of hypoacetylated EMRs (**Fig 7F**). In contrast, hypoacetylated PMRs annotated to genes implicated in apoptosis, differentiation, polyamine metabolism, and bone morphogenetic protein (BMP) and G-protein–coupled signaling, and they contained recognition sites for TEAD, MYOG, BTB domain and CNC homolog (BACH), and JUN (**S4H Fig,** bottom and **S4I Fig,** right). Thus, differential enhancer analysis predicted exercise to direct MEF, SIX, NRs, and bZIPs to tissue remodeling and metabolic gene enhancers, whereas PGC1α was anticipated to coactivate ERR and MEF2 at sites along lipid metabolic genes.

To complement our histone mark–based analysis, we performed ATAC-seq in Quad skeletal muscles and identified peaks from sedentary control (47,326 peaks), exercise-trained (47,346 peaks), and mTg mice (28,711 peaks). Although we identified few differences between conditions with respect to open chromatin regions (only 280 differential peaks between exercise and control or 127 peaks between mTg and control), we further analyzed our data sets to detect small stretches of DNA protected from transposase cleavage due to transcription factor binding, which are known as DNA footprints. Using the Hmm-based identification of transcription factor footprints (HINT)-ATAC computational pipeline [36], we identified footprints in control versus either Ex or mTg muscle and calculated activity scores by measuring footprint depths and the total number of reads in the flanking regions. Consistent with the hypoacetylation observed in Ex tissue, significant activity scores were all reduced by exercise including AT-rich interaction domain (ARID), E2F transcription factors (E2F), homeobox, and to a lesser extent NR (nuclear recptor subfamily 2 group F member 6 [NR2F6], retinoic acid receptor beta [RARβ]) motifs (**S5A Fig**). In contrast, significant activity scores were all higher in mTg muscle and included ARID and many homeobox motifs (**S5B Fig**). Although footprinting produced a number of different motifs than our EMR and PMR H3K27ac-based analyses, it is notable that many transcription factors do not leave measurable footprints on DNA and thus limit this approach [37].

### Exercise and PGC1α convergently control metabolic gene expression

**Fig 8:**
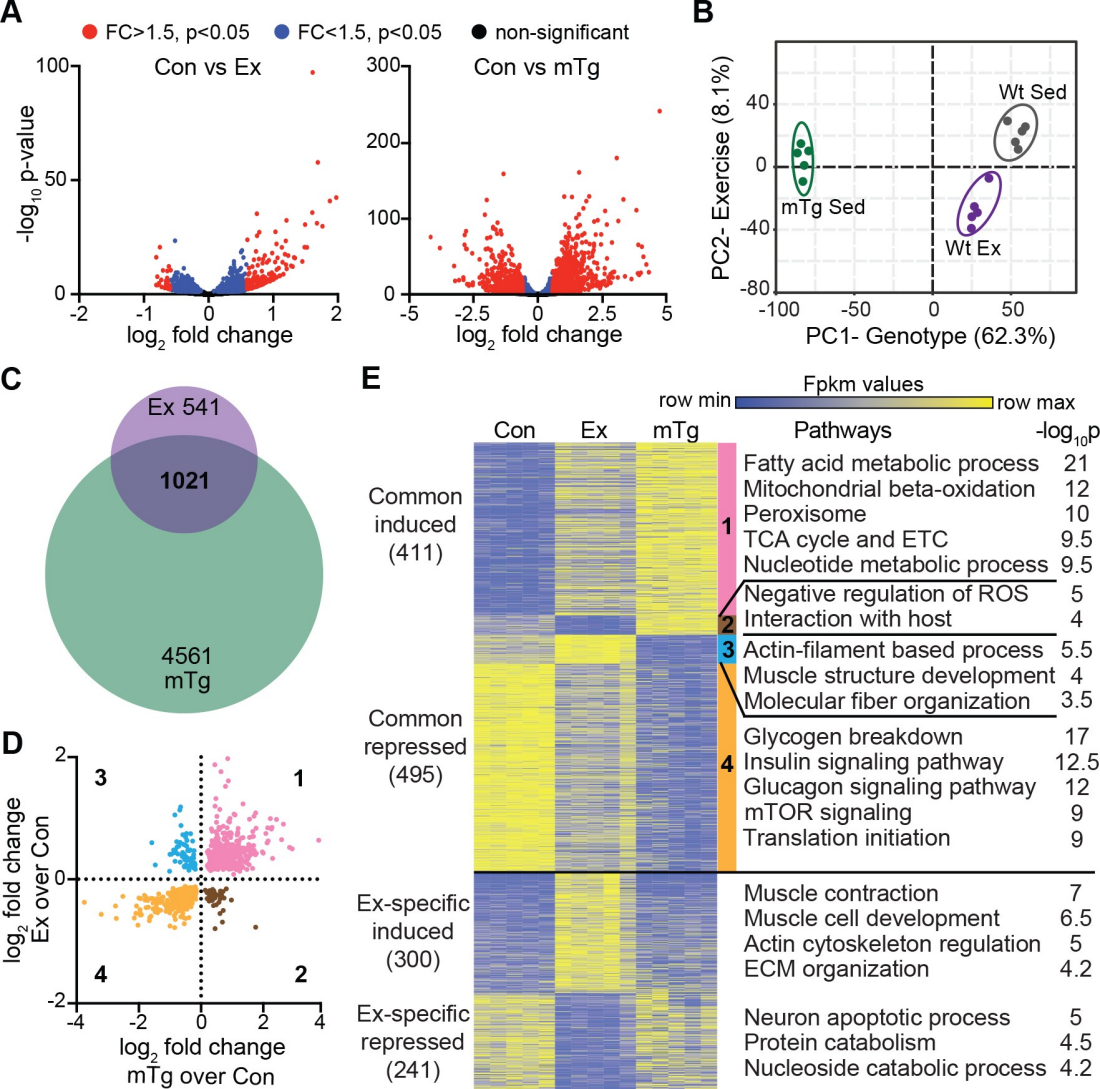
Exercise and *Pgc1a* overexpression convergently control gene expression. (A) Volcano plots showing log_2_ FC in gene expression in Ex (left) or mTg (right) compared with Con quadriceps. Red represents absolute FC > 1.5 with an adjusted *p*-value < 0.05, blue represents *p*-value < 0.05, and black represents nonsignificant genes (*n* = 5/group). (B) Principal component analysis of all significantly altered genes (*p* < 0.05) in Ex or mTg compared with control. (C) Venn diagram showing transcriptional overlap in Ex and mTg. (D) Quadrant plot of log_2_ FC in gene expression in Ex/Con versus log_2_ FC in mTg/Con. (E) Heatmap of K-means clustering of transcriptomic profiles for differentially expressed genes. The number of genes in each cluster is in parenthesis. Each cluster, indicated by a vertical color bar, corresponds to quadrant colors from the previous panel. Two clusters were unique to Ex-regulated genes (bottom). Pathways enriched in each cluster and corresponding −log_10_ *p*-values are shown on the right. Numerical values for all panels are available in S1 Data. Con, control; ECM, extracellular matrix; ETC, electron transport chain; Ex, exercised; FC, fold change; Fpkm, Fragments Per Kilobase of transcript per Million mapped reads; mTg, muscle-specific *Pgc1a* transgenic; mTOR, mammalian target of rapamycin; PC1, principal component 1; PC2, principal component 2; *Pgc1a*, peroxisome proliferator–activated receptor gamma, coactivator 1 alpha; ROS, reactive oxygen species; Sed, sedentary; TCA, citric acid cycle; Wt, wild-type.

To understand the transcriptional impacts of the Ex- and mTg-induced epigenomic programs, we used mRNA-seq of quadriceps to comprehensively analyze gene expression in these models compared with sedentary wild-type controls. Ex mice exhibited 779 significantly induced and 783 suppressed genes (*p*-value < 0.05) (**Fig 8A,** left). mTg mice demonstrated many more changes in expression, with 2,843 up-regulated and 2,739 down-regulated genes (**Fig 8A,** right). Principal component analysis confirmed that genotype was the dominant variable, whereas exercise was secondary, and experimental replicates from each condition clustered tightly (**Fig 8B**). Nearly two-thirds of exercise-regulated transcripts were also impacted by *Pgc1a* overexpression (**Fig 8C**), and expression changes positively correlated among 89% (906/1,021) of these between the two models (**Fig 8D**). Genes altered similarly in Ex and mTg muscles were implicated in glucose, nucleotide, and particularly, fatty acid metabolism, as well as insulin and mammalian target of rapamycin (mTOR) signaling (**Fig 8D and 8E,** clusters 1 and 4). However, 115 genes were oppositely controlled by Ex and mTg and annotated to genes with functions in muscle structure and regulation of reactive oxygen species (**Fig 8D and 8E**, clusters 2 and 3). Additionally, 541 transcripts were altered by exercise but not impacted by *Pgc1a* overexpression. These exercise-exclusive targets were associated with muscle development and contraction, extracellular matrix organization, and protein and nucleotide metabolism (**Fig 8E**). In summary, despite eliciting highly divergent epigenomic profiles, exercise and *Pgc1a* overexpression similarly regulate transcription among metabolic genes, but exercise is distinct in its control of tissue-remodeling genes.

To assess the functional consequences of exercise- and PGC1α-induced transcription, we performed immunohistochemical analysis of skeletal muscle. In line with the similar oxidative transcriptomic profiles of Ex and mTg mice, succinate dehydrogenase staining was increased in both models compared with controls, although staining was considerably more ubiquitous in mTg animals (**S6A Fig**). Additionally, we interrogated *Myh* isoforms using antibodies to MYH4 (type IIb), MYH1 (type IIx), MYH2 (type IIa), and MYH7 (type I) (**S6B Fig**). Glycolytic type IIb fibers were diminished by 14% and 20%, whereas more oxidative type IIa fibers were increased by 103% and 32% in Ex and mTg mice, respectively, compared with controls. However, type I and type IIx content was selectively increased in mTg mice by 73% and 141% compared with controls but was not substantially changed in Ex muscle. Hence, exercise or *Pgc1a* overexpression promote oxidative metabolism and myofiber switching, but myofiber profiles are distinct in these models.

### Integrative *cis*-regulatory analysis predicts causal factors for muscle adaptation

**Fig 9:**
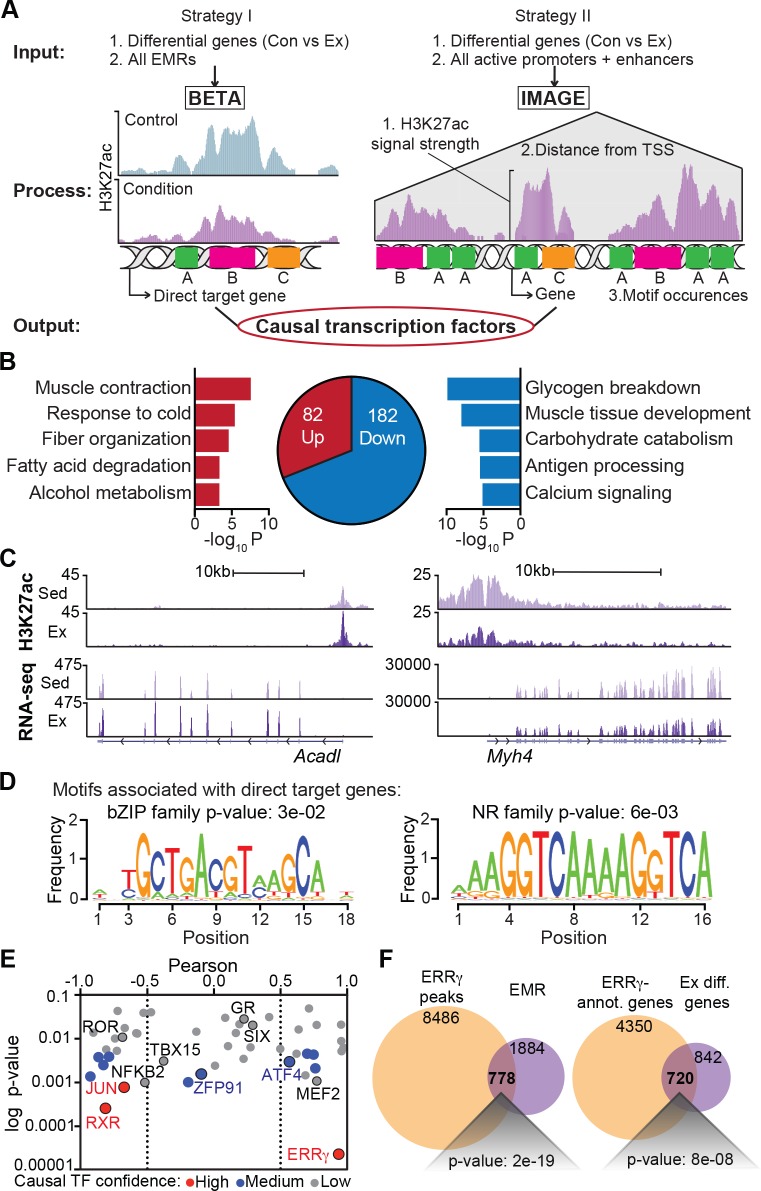
Integrative *cis*-regulatory analysis predicts causal factors for exercise-induced reprogramming. (A) Flowchart depicting the two approaches (BETA and IMAGE) used to perform integrative transcriptomic and epigenomic analysis. (B) Gene ontology analysis of differentially marked H3K27ac peaks (EMRs) associated with differentially expressed genes in Ex Quad versus Con identified by BETA. Directly up-regulated (red) and down-regulated (blue) gene numbers and ontologies are shown. (C) Representative UCSC browser tracks of H3K27ac (top) and RNA-seq (bottom) in Sed Quad (light purple) and Ex Quad (dark purple). (D) Motifs associated with the directly up-regulated (left) and down-regulated (right) genes identified by BETA. (E) Log *p*-value versus Pearson correlation plotted for causal transcription factors identified by IMAGE. Red = high-confidence factors, blue = medium-confidence factors, and gray = low-confidence factors. (F) Venn diagram showing overlap of EMRs with ERRγ peaks (left) and overlap of genes annotated near ERRγ binding sites with exercise-regulated genes (right). Numerical values for all panels are available in S1 Data. *Acadl*, Acyl-CoA dehydrogenase long chain; BETA, Binding and Expression Target Analysis; bZIP, basic leucine zipper; Con, control; EMR, exercise-modified region; ERR, estrogen-related receptor; Ex, exercised; H3K27ac, histone 3 lysine 27 acetylation; IMAGE, Integrated analysis of Motif Activity and Gene Expression; *Myh*, myosin heavy chain; NR, nuclear receptor; Quad, quadriceps femoris; RNA-seq, RNA sequencing; Sed, sedentary; TSS, transcription start site; UCSC, University of California, Santa Cruz.

We next integrated our transcriptomic and epigenomic modification analyses to gain insight into the transcription factors responsible for skeletal muscle adaptation to exercise. To do so, we used two complementary approaches (**Fig 9A**). In the first, we tested the extent to which EMRs, the regions exhibiting the greatest changes in H3K27ac modification, could account for exercise-induced changes in gene expression using the Binding and Expression Target Analysis (BETA) pipeline [38]. We found that among genes that were transcriptionally altered by exercise and located within 100 kb of an EMR, 82 genes were up-regulated in the vicinity of these sites, whereas 182 were down-regulated (**Fig 9B**). The predominance of repressed genes corresponded to a negative regulatory potential for EMRs (*p*-value = 2 × 10^−5^), consistent with the overall impact of exercise to cause hypoacetylation at these sites. Ontologies for EMR-associated up-regulated targets, such as Acyl-CoA dehydrogenase long chain (*Acadl*) (**Fig 9C**, left), included genes implicated in muscle contraction, fiber organization, and fatty acid degradation (**Fig 9B**). Ontologies for EMR-associated down-regulated genes, such as *Myh4* (**Fig 9C,** right), included glycogen breakdown, muscle development, and carbohydrate catabolism (**Fig 9B**). Consistent with our motif analysis for all EMRs (**Fig 7B and 7E**), the subset of EMRs associated with altered gene expression by BETA was enriched in motifs for bZIPs and NRs (**Fig 9D**). However, the majority of genes regulated by exercise lacked EMRs, indicating that dynamic changes in H3K27ac only captured a subset of transcriptionally relevant regulatory events at nearby enhancers and promoters.

To more fully reveal regulators of exercise-induced adaptation, our second approach applied a recently developed method known as Integrated analysis of Motif Activity and Gene Expression (IMAGE) [39], which uses a machine-learning strategy. IMAGE identifies the contribution of enhancer and promoter motifs to all transcriptional changes based on the distance-weighted sum of motif occurrences in all predicted target gene regulatory regions (**Fig 9A**, right). To implement IMAGE, we defined active regulatory regions as sites exhibiting H3K27ac and H3K4me2 peaks or H3K27ac peaks and accessible chromatin based on ATAC-seq. Motifs within these 33,067 active regulatory regions were then analyzed for their impact on exercise-induced transcription. IMAGE analysis identified 50 transcription factors with significant predicted roles in exercise programming (**S1 Table**). Among these, 13 emerged as causal factors with high or medium confidence. The nuclear hormone receptors ERR and retinoid X receptor (RXR) along with JUN were top scoring, followed by forkhead box protein O4 (FOXO4), NFYB, zinc finger protein 91 (ZFP91), TEAD, signal transducer and activator of transcription 5A (STAT5A), activating transcription factor 4 (ATF4), nuclear factor kappa B subunit 2 (NFKB2), MEF2A, cytoplasmic polyadenylation element binding protein 1 (CPEB1), T-box 15 (TBX15) (**Fig 9E**). Compared with IMAGE analysis in mTg mice (**S2 Table**), NFKB2, ZFP91, ATF4, and TBX15 were uniquely identified as causal factors in exercise. To verify motif predictions from our analyses, we compared genome-wide DNA binding sites of transgenically expressed *Errg* in Quad [40] to EMRs. Nearly 30% (778/2,662) of EMRs colocalized with ERRγ bound sites (**Fig 9F,** left), in keeping with the strong enrichment of ERR/NR binding sites predicted in our motif analysis. Similarly, transgenic ERRγ binding was detected at 46% (720/1,562) of exercise-regulated genes (**Fig 9F,** right). Hypergeometric tests confirmed that the ERRγ enrichment at EMRs and exercise-regulated genes is highly enriched relative to its binding at all Quad H3K27ac-marked regions (*p*-value = 2 × 10^−19^) or all Quad-expressed genes (*p*-value = 8 × 10^−8^), respectively. These findings attest to the role for ERRs in skeletal muscle adaptation and validate our analytical methods to reveal the epigenomic regulators of exercise.

## Discussion

Muscle differentiation and adaptation have been extensively studied, but epigenome-level analyses of skeletal muscle in vivo are lacking. Using ChIP-seq of H3K4me2 and H3K27ac, we have established maps for regulatory regions and their activities in different muscle groups. These profiles pointed to MEF2, SIX1, NUR77, ERR, and NFAT as key determinants of developmental myofiber specification. Integrative regulatory region and gene expression analysis also enabled us to predict causal effectors of skeletal muscle adaptation, revealing several well-known factors, such as ERR and RXR nuclear hormone receptors, JUN, MEF2, SIX, and many less-characterized mediators of exercise-induced programming.

Our findings indicate that the tissue environment plays a key role in shaping skeletal muscle enhancer networks. Muscles exhibit a substantial number of distinguishing regulatory features, including enhancers and SEs suited to their specific contractile properties and functional demands. Remarkably, muscle regulatory regions are uncorrelated with those identified in differentiated C2C12 MTs. It has long been recognized that gene regulation in vitro and in vivo are discrepant, likely because of genotypic alterations in immortalized cells and lack of developmental, mechanical, or environmental stimuli that can change the activities of transcription factors [41]. Reminiscent of our findings, adipocytes show only moderate correlation between histone-modification landscapes of in vitro differentiated 3T3L1 cells and primary cells in vivo [42]. Similarly, macrophages demonstrate distinct enhancer repertories in vitro and in vivo or between different tissue-resident macrophage populations [9]. Thus, our work provides an important resource to advance understanding of skeletal muscle gene regulation, which has not been fully addressed in prior research.

A number of transcription factors implicated in genetic studies of muscle development and fiber-type specification were identified in our analysis, providing new insight into their epigenomic coordination within different muscle groups. MEF2 sites emerged as the most statistically enriched among skeletal muscle active regulatory regions and subsets of muscle group–specific sites, highlighting the critical role for MEF2 proteins in myogenesis and myofiber programming [27]. SIX motifs were concentrated in hyperacetylated regulatory regions of quadriceps compared with Sol muscle. SIX proteins, particularly SIX1, are also recognized for their roles in early myogenesis and the activation of fast-twitch fiber–specific genes to promote slow-to-fast fiber transitions [43,44]. Likewise, motifs for NUR77 were overrepresented in quadriceps, which is expressed predominantly in glycolytic tissue and controls genes involved in glucose utilization [45]. In contrast, hyperacetylated H3K27 sites in Sol were enriched in motifs for NFATs and ERRs, which have been shown to activate slow myosin expression and contribute to the establishment of oxidative fibers [29,30,46]. Thus, SIX and NUR77 or NFATs and ERRs appear to play key roles in glycolytic versus oxidative myofiber development, respectively, whereas MEF2 regulation contributes to both.

PGC1α-driven reprogramming of skeletal muscle enhances skeletal muscle oxidative metabolism and endurance, but the extent to which it mimics the signaling effects of exercise has been unclear. At the level of the epigenome, we found thousands of H3K27ac signal differences between these models. PMRs were associated with hyperacetylation and markedly enriched in MEF2 and ERR motifs, whereas EMRs were surprisingly hypoacetylated and contained an abundance of MEF2 and NR half sites. The basis for the dichotomy of H3K27ac signals in EMRs and PMRs, particularly in light of their similar motifs, remains an unresolved question. It is possible that MEF2 could favor the nuclear retention of HDACs in the setting of chronic training, in contrast to the dismissal of class II HDACs observed with acute exercise [47]. Alternatively, the composition of NR complexes on EMRs and PMRs may be different. In support of this, studies have shown that ERRγ promotes oxidative muscle remodeling independently of PGC1α and β through an unknown assembly of cofactors [40]. We found that ERRγ binds to nearly 30% of EMRs or 26% of PMRs, which likely represent sets of PGC1-independent or PGC1-containing ERR complexes, respectively. Thus, in spite of similar changes in transcription from exercise and *Pgc1a* overexpression, their epigenomic signatures are highly distinctive and suggest the participation of different factors in muscle reprogramming.

The unique epigenomic and transcriptional profiles identified in Ex and mTg mice may also be influenced by timing and intrinsic differences in our models, as well as limitations in our analysis. Exercise transiently up-regulates and activates PGC1α [48–51], whereas *Pgc1a* transgenesis results in its constitutive overexpression. Our interrogations of exercise-trained mice were performed within 6 hours or 72 hours of their last bout of wheel running, when *Pgc1a* levels were not elevated, whereas mTg mice exhibited chronic elevations in *Pgc1a*. Thus, timing of analysis and differing levels of PGC1α in Ex versus mTg mice may have contributed to divergence in epigenomic signals between the two models, especially because H3K27ac has a half-life of only approximately 2.3 hours [52]. Whereas H3K27ac modification has received considerable attention as a marker for regulatory region activity [10,11,13,14], other modifications can alternatively reflect activity in the absence of H3K27ac, including histone 4 lysine 16 acetylation (H4K16ac), histone 3 lysine 64 acetylation (H3K64ac), or histone 3 lysine 122 acetylation (H3K122ac) [53,54], which were not analyzed. Thus, it is possible that further distinctions or similarities in enhancer activities were not captured. Work to determine the three-dimensional organization of skeletal muscle chromatin will be an important future step to further define and assign the regulatory regions identified herein to their associated target genes, particularly because only half [55] to two-thirds [56] of regulatory elements have interactions with the nearest expressed gene, and many elements mediate long-range interactions across hundreds of kilobases. Such structural insight may also help to reconcile how Ex and mTg mice impart relatively similar effects on gene expression, despite their distinctive EMRs and PMRs.

Many transcriptional regulators have been linked to muscle adaptation, yet their relative contributions have been unknown. We have delineated these and identified undescribed or minimally characterized factors in exercise-directed remodeling. Enhancer activity and IMAGE-based analyses each revealed ERR, RXR, JUN, and MEF2 motifs as top scoring, attesting to the primacy of NRs and downstream effectors of MAPK and calcineurin signaling in muscle transcriptional remodeling [57–60]. Additionally, SIX proteins were identified using both analysis strategies, pointing to an underappreciated role for these developmental regulators in adaptive reprogramming [43,44]. Many other factors were inferred by IMAGE analysis. For example, the glycolytic fiber regulator TBX15 was negatively associated with chronic exercise training, whereas the slow myofiber regulator TEAD was positively correlated [61,62]. The noncanonical NFKB2, which has been linked to oxidative metabolism in muscle, was also identified, along with its activator ZFP91 [63,64]. Multiple proteins associated with muscle atrophy or hypertrophy were inferred, including STAT5A, FOXO4, MAFB, and ATF4. In addition, the GDNF inducible zinc finger protein 1 (GZF1) and heat shock transcription factor 4 (HSF4) repressors and NFYB were identified, which have little known function [65,66]. Some of these putative transcriptional effectors of exercise, including MEF2, SIX, and TEAD, were also identified in a motif-based analysis of differentially methylated DNA positions in muscle from humans subjected to chronic training [67]. Hence, our integrative epigenomic analysis points to conserved involvement of several of the aforementioned transcription factors in skeletal muscle regulation with exercise, and it differs entirely from predictions based solely upon gene expression analysis [68].

Health improvements associated with exercise are multifaceted, so understanding skeletal muscle adaptation and its molecular basis could provide insight to therapy for a variety of diseases. PGC1α and ERR-driven pathways are beneficial in the context of many skeletal muscle maladies including atrophy [69,70], mitochondrial dysfunction [71], ischemia [35,72], age-related deterioration [73], amyotrophic lateral sclerosis [74], and muscular dystrophy [75–77]. Yet studies of PGC1α or ERR have produced mixed effects with respect to some signaling and health enhancements associated with exercise. Genetic activation of PGC1α or ERR has been reported to have negative [78], neutral [79,80], or positive [81] impacts on insulin sensitivity in various experimental contexts. Although genetic loss of *Pgc1a* impairs exercise-induced angiogenesis, it does not impact exercise-induced fiber-type transformation [82,83]. Even mice lacking both PGC1α and PGC1ß in skeletal muscle do not have significantly altered fiber-type profiles or insulin sensitivity [84]. Accordingly, further studies are warranted to establish such roles for several of the lesser-known transcription factors identified here. Additionally, future integration of our data sets with other “omic” studies [20] will be a promising approach to unravel the complex impact of exercise on cell-intrinsic signaling and whole-body physiology.

## Methods

### Ethics statement

All mouse work was approved by the Northwestern University Institutional Animal Care and Use Committee (IACUC) (protocol number IS00005071). All animal care and procedures adhered to the United States Public Health Service (USPHS) regulations and applicable federal and local laws. All mice were euthanized using CO_2_ inhalation followed by cervical dislocation.

### Animals

Male C57BL/6J mice (7–8 weeks old) were purchased from Jackson Laboratories and maintained with free access to chow and water. For voluntary exercise studies, mice were randomly assigned to individual cages with or without free access to wheels at 8 weeks of age for 4 weeks. Ex mice were harvested 4–6 hours after the last bout of exercise. Detraining mice were harvested 72 hours after their last bout of exercise. Skeletal muscle–specific mTg mice were generously provided by Dr. Bruce Spiegelman. Male transgenic and wild-type littermates were used for all experiments.

### ChIP followed by sequencing or qPCR

ChIP was performed as previously described [85], with modifications. For histone-modification ChIP-seq, a single quadriceps muscle from each animal was used for each sample, whereas EDL, Sol, or Dia muscles from three animals were pooled to make each biological sample. Muscles were harvested, rinsed in PBS, and cross-linked at room temperature for 30 minutes in 1% formaldehyde. After quenching with 125 mM glycine, cross-linked material was rinsed twice with cold PBS and frozen at −80°C until further processing. Cross-linked material was incubated in 20% trypsin in PBS/EDTA buffer for 30 minutes on ice. Then, the pellet was homogenized in a buffer containing 67 mM mannitol, 50 mM Tris/HCl, 50 mM KCl, 10 mM EDTA, and 0.2% BSA adjusted to a pH of 7.4. The homogenized material was spun at 700*g* for 10 minutes, and the pellet was resuspended in buffer containing 1% SDS, 10 mM EDTA, and 50 mM Tris, and then it was passed through 70-μm Filcons (BD Biosciences) to purify nuclei in the flowthrough. The isolated nuclei were sonicated for 10 cycles (30 seconds on, 30 seconds off) using a Diagenode Bioruptor to shear chromatin into 200–1,000-bp fragments. Fixed, sonicated chromatin was incubated with antibody against H3K4me2 (AbCam #32356) or H3K27ac (Active Motif #39133) overnight. Antibody complexes were precipitated with anti-rabbit IgG paramagnetic beads (ThermoFisher). Chromatin was de-cross-linked, purified, and used to generate libraries using KAPA DNA Library Preparation kits (Kapa Biosystems). During library preparation, adapter-ligated ChIP DNA was size selected to obtain inserts of 200–500 bp using a PippenHT (Sage Science). Libraries were assessed by both a Bioanalyzer (Agilent) and qPCR-based quantification (Kapa Biosystems) and sequenced on an Illumina NextSeq 500 using 75-bp single-end reads. Read counts are listed in **S3 Table**.

For transcription factor (TF) ChIP, when performing sequencing, quadriceps from five animals or Sol from 10 animals were pooled to make one biological sample, whereas for qPCR validation, quadriceps from two animals or Sol from five animals were pooled to make one sample. Muscles were harvested, minced, and processed using a Polytron tissue homogenizer in buffer (0.15 mM NaCl, 0.05 mM EDTA [pH 7.5], 0.5 mM Tris, 0.5% NP40, 1% Triton X-100). The homogenate was then dounced (approximately 10 strokes), and serial filtering from 150 μm to 30 μm was performed. Filtrate was then spun at 600*g* for 10 minutes, and the isolated nuclei were cross-linked at room temperature, first with 2 mM disuccinimidyl glutarate for 20 minutes and then with 1% formaldehyde for 5 minutes. After quenching with 125 mM glycine, cross-linked nuclei were rinsed with cold PBS and then sheared in a buffer containing 1% SDS, 10 mM EDTA, and 50 mM Tris for six cycles (30 seconds on, 30 seconds off) using a Diagenode Bioruptor to shear chromatin into 200–1,000-bp fragments. Fixed, sonicated chromatin was incubated with 7.5–10 μg of antibody against MED1 (Bethyl Labs #A300-793A) or NFATc1 (Millipore #MABS409) and 3 μg of SIX1 (Proteintech #10709-1AP), c-JUN (Cell Signaling #9165), or normal rabbit IgG (Santa Cruz #2027) overnight. Antibody complexes were precipitated with anti-rabbit or anti-mouse IgG paramagnetic beads (ThermoFisher) for sequencing or with protein A agarose beads (Millipore) for qPCR. Chromatin was de-cross-linked, and DNA was isolated using MinElute PCR purification columns (Qiagen). Library preparation for MED1 and NFAT ChIP samples was performed as described above. ChIP qPCR assays for SIX1 and c-JUN were performed in technical duplicates. Primer sequences for ChIP qPCR are listed in **S4 Table.**

### ATAC-seq

Muscles were dissected, frozen in liquid nitrogen, and pulverized with a Covaris CP02 cryoPREP. Approximately 30 mg of powdered frozen tissue was transferred to 1.5-ml tubes and resuspended in ice-cold nuclei lysis buffer containing 20 mM Tris-HCl, 50 mM EDTA, 5 mM spermidine, 0.15 mM spermine, 0.1% mercaptoethanol, and 40% glycerol (pH 7.5). Samples were filtered through Miracloth (EMD Millipore) and centrifuged at 1,100*g* for 10 minutes at 4°C. Nuclear pellets were subjected to transposition and library preparation [15] and sequenced on an Illumina NextSeq 500 using 42-bp paired-end reads. Separate ATAC-seq experiments were performed to compare open chromatin across muscle groups (EDL, Quad, Sol, Dia) and across muscle-remodeling conditions (sedentary, Ex, and mTg Quad).

### Analysis of ChIP-seq and ATAC-seq data

For ChIP-seq, raw sequence reads were aligned to the mouse reference genome (mm10) using Bowtie version 1.1.2 [86] using “-m 1” and “—best” options to ensure reporting of uniquely mapped reads. For ATAC-seq, Fastq files were aligned using Bowtie2 (version 2.2.6) and default settings. Previously published data sets of H3K27ac ChIP-seq (SRP012465 and SRP002800) and ATAC-seq (SRP093753) in differentiated C2C12 MTs and ERRγ ChIP-seq (SRP110311) in *Errg* transgenic muscle were reanalyzed in the same way for comparisons. Tag directories were generated using “makeTagDirectory” using the -tbp 1 option to limit the number of reads starting at the same position to 1.

Further analysis of ChIP-seq and ATAC-seq was performed with HOMER [87]. Peaks for individual replicates were identified using the “findPeaks” command, specifying “-style histone” for histone ChIP-seq or “-style factor” for TF ChIP-seq and ATAC-seq. To generate high-confidence peak lists across both replicate samples for downstream analysis, ChIP-seq peaks were identified using the HOMER “getDifferentialPeaksReplicates.pl” command, specifying “-style histone.” This command uses DESeq2 and identifies peaks that pass 2-fold enrichment and false discovery rate (FDR) < 0.05 cutoffs. SE peaks were also identified from H3K27ac (FDR = 0.001), ATAC (FDR = 0.001), and Med1 (FDR = 0.05) data sets using the “findPeaks” command, specifying “-style super.” High-confidence SEs were defined as peaks that were found in at least two out of these three ways for finding SEs. Peaks were annotated to nearest genes using “annotatePeaks.pl” and classified as occurring at promoter (−1 kb to +100 bp from TSS), intragenic (coding exon, intronic, 3′UTR, 5′UTR, TTS, noncoding exon), or intergenic locations. The “makeUCSCfile” command was used to generate UCSC browser tracks. HOMER’s “mergePeaks” was used to compare different peak sets with option “-d given” to obtain literal overlaps in peak regions.

To generate the tag density scatter plots and histograms, tags were quantified using HOMER’s “annotatePeaks.pl” command, with either “-size 1000” option or “-size 6000 -hist 10” options, for scatter plots and histograms, respectively. H3K4me2 and H3K27ac distribution plots were generated by quantifying tags using HOMER’s “annotatePeaks.pl” command with “-size 6000 -hist 25 or 50” and specifying the “-ghist” option, whereas ATAC-seq distribution plots were generated using the same command but with “-size 4000.” Morpheus [88] was used for heatmap visualization, hierarchical clustering, and Spearman correlation similarity matrix analysis. BoxPlotR [89] was used to generate boxplots of H3K4me2 and H3K27ac or NFAT tags in different conditions by providing tags quantified using HOMER’s “annotatePeaks.pl” command, with “-size 1000” or “-size 500” option, respectively. Venn diagrams were generated using Intervene [90].

HOMER's “getDifferentialPeaksReplicates.pl” command was also used to find peaks that were differentially methylated or acetylated between any two muscle groups or in quadriceps of the two different models compared with respective controls. For these analyses, peak lists combining all peaks from the comparison conditions were assembled. One condition was used as the target, and the other was used as background. The option “-balanced” was used to allow data to be quantile normalized. To characterize enriched motifs near differentially acetylated peaks, we used HOMER’s “findMotifsGenome.pl” command to scan the entire peak by using “-size given” with standard background. Gene ontologies for ChIP-seq data were generated using GREAT [91] by annotating ChIP-seq peaks to two nearby genes.

ATAC-seq data were analyzed for transcription factor footprints using the Regulatory Genomics Toolbox application HINT [36] using the “-atac-seq” and “-paired end” parameters. Replicates for control and exercise data sets were pooled to increase read depth and then subjected to rgt-hint analysis. Footprint motifs and differential motif occupancy were then assessed with the “rgt-motifanalysis matching” and “rgt-hint differential” commands.

Integrative ChIP and RNA-seq analysis was conducted using BETA [38] and IMAGE [39]. For both of these tools, we provided differentially expressed genes found by DESeq2 along with either the differentially acetylated peak list or all active regulatory regions (defined by presence of H3K27ac peak along with either H3K4me2 or ATAC peak) as inputs and used default parameters to execute the programs.

### RNA isolation and sequencing

Muscles were harvested and snap frozen in liquid nitrogen. Tissues were homogenized in 1 mL TriZol (Qiagen) using the Mo Bio Powerlyzer. RNA was isolated using RNeasy columns (Qiagen). RNA quality was assessed using a Bioanalyzer (Agilent) to ensure RIN scores > 7.0 prior to library construction. Sequencing libraries were constructed using Illumina TruSeq Stranded mRNA sample prep kits. Libraries were quantified using both a Bioanalyzer (Agilent) and qPCR-based quantification (Kapa Biosystems), and they were sequenced on an Illumina NextSeq 500 instrument using 75-bp single-end reads.

### Analysis of RNA-seq data

Sequenced reads were aligned to the mm10 reference mouse genome using STAR [92], and differentially expressed RNAs were identified using DESeq2 [93] (FDR-adjusted *p*-value < 0.05) via the RNA-seq Alignment (v1.1.1) DESeq2 (v1.1.0) BaseSpace application (Illumina). Direct comparisons were made between control and Ex animals, as well as between control and mTg animals to generate lists of differentially expressed genes. Volcano plots were generated using GraphPad Prism 7.0 (GraphPad Software). Principal component analysis was performed using a web-based tool, ClustVis [94]. Morpheus [88] was used for K-means clustering and heatmap visualization by giving the Fpkm values for differential genes common to both mTg and Ex muscle compared with control tissues as well as genes that are uniquely differential in exercise versus control as the input. Gene ontology analysis was performed on differential genes using Metascape [95]. Enrichment analysis included terms from Reactome Gene Sets, GO Biological Processes, and KEGG Pathways.

Previously published data sets of RNA-seq were reanalyzed in the same way as our own primary expression data. For comparisons in EDL, Quad, Sol, and Dia of C57BL/6J mice, sequence read archive SRP110541 was used [22]. Fpkm values for genes in the four muscle groups were used to perform clustering analysis using Morpheus [88]. Differential gene expression between Quad and Sol was calculated using DeSeq2 with default settings, prior to correlation with H3K27ac and ATAC-seq. This was done by using HOMER’s “annotatePeaks.pl” command to quantify H3K27ac or ATAC tags in Quad and Sol with “-size 50000” option at TSSs of genes identified as significantly up-regulated in Quad or Sol compared with the other group. Previously published RNA-seq data in differentiated C2C12 MTs (SRP161899) [96] was reanalyzed in the same way for comparisons. Differential gene expression between Quad and MT was calculated using EdgeR with default settings, prior to correlation with H3K27ac or ATAC. Pathway analysis at acetylated regions that annotate to differential genes was performed using GREAT [91].

### qPCR

cDNAs were synthesized from RNA using iScript cDNA synthesis kits (Bio-Rad). Quantitative real-time PCR analysis was performed with SYBR Green Master Mix (Bio-Rad) and analyzed using a Bio-Rad CFX384 Touch Real-Time PCR System. Expression was determined using the relative standard curve method and normalized to the housekeeping gene *36b4*. Primer sequences are listed in **S5 Table**.

### Histology

Muscle was frozen in OCT embedding compound and cut with 10-μm transverse cryo-sections taken at midbody at least 150 μm apart. For immunostaining, muscle sections were fixed with ice-cold acetone, washed with PBS, and incubated in blocking solution (10% FBS, 0.1% Triton X in 1× PBS) for 1 hour at room temperature. Next, primary antibodies diluted in blocking solution were added and incubated overnight at 4°C. The following mouse monoclonal antibodies from Developmental Studies Hybridoma Bank were used for fiber staining: BA-F8, type I (1:10 dilution); SC-71, type 2A (1:30 dilution); and BF-F3, type 2B (1:10 dilution). The next day, slides were washed with PBS and incubated with secondary antibodies for 1 hour at room temperature, washed, and mounted with Prolong Antifade Diamond. For SDH staining, slides were thawed and covered in incubating solution (50 mM sodium succinate, 0.5 mg/ml nitroblue tetrazolium) for 90 minutes at 37°C. Slides were then incubated at 23°C for 15 minutes in formal calcium (40% formaldehyde, 10% CaCl2), washed in twice-distilled H_2_O, and mounted with Prolong Antifade Diamond (Invitrogen). All slides were imaged on an Olympus VS120 slide scanner.

### Statistical analysis

We used Microsoft Excel, GraphPad Prism 7.0 (GraphPad Software), and Sigmaplot 11.0 to perform the statistical analyses. Student’s unpaired *t* test or two-way ANOVA followed by multiple comparisons analysis with Tukey’s correction was used to compare qPCR gene expression between two or multiple groups, respectively. Spearman correlation analysis was performed on normalized ChIP-seq tag counts for all pairwise comparison scatterplots. One-way ANOVA with Tukey’s correction was performed on normalized ChIP-seq tag counts to compare quadriceps versus Sol and Ex or mTg with control. All sample sizes and *p*-values are listed in the figure legends.

### Accession numbers

All RNA-seq, ChIP-seq, and ATAC-seq data are deposited in GEO SuperSeries accession #GSE123879.

## Supporting information

S1 Fig Genomic localization of enhancer regions and their correlation across muscle groups. (A,B) Scatterplots of normalized H3K4me2 (A) and H3K27ac (B) tag counts for individual ChIP-seq replicates in the four muscle groups. Correlation coefficient (r_s_) was calculated for each scatterplot. (C) Genomic distributions of H3K4me2 (left) and H3K27ac (right) peaks across the four muscle groups in promoter, intragenic, and intergenic regions. (D,E) Scatterplots of normalized H3K4me2 (D) and H3K27ac (E) tag counts at genomic regions marked by H3K4me2 and H3K27ac tags in pairwise comparisons of four different muscle groups. Correlation coefficient (r_s_) was calculated for each scatterplot (*n* = 2/group). (F) Heatmap of motifs enriched in H3K27ac peaks for EDL, Quad, Sol, and Dia, with *p*-values represented in color. Numerical values for all panels are available in S12 Data. ChIP-seq, chromatin immunoprecipitation sequencing; Dia, diaphragm; EDL, extensor digitorum longus; H3K4me2, histone 3 lysine 4 dimethylation; H3K27ac, histone 3 lysine 27 acetylation; Quad, quadriceps femoris; Sol, soleus.
(TIF)

S2 Fig Gene expression levels correlate with H3K27ac as well as ATAC-seq signals between muscle groups. (A) Similarity matrix for Fpkm values from RNA-seq data for EDL, Quad, Dia, and Sol. Pearson correlation coefficient (r) is represented in color; *n* = 4/group. (B) H3K27ac tags within 50 kb around the TSS for genes differentially expressed (log2 fold change > 1.5) in Quad > Sol (left) or Sol > Quad (right); *n* = 2/group, ***p < 0.001. (C) ATAC-seq tags within 50 kb around the TSS for genes differentially expressed (log2 fold change > 1.5) in Quad > Sol (left) or Sol > Quad (right); *n* = 2/group, **p < 0.01, ***p < 0.001. Numerical values for all panels are available in S12 Data. ATAC-seq, assay for transposase-accessible chromatin sequencing; Dia, diaphragm; EDL, extensor digitorum longus; Fpkm, Fragments Per Kilobase of transcript per Million mapped reads; H3K27ac, histone 3 lysine 27 acetylation; RNA-seq, RNA sequencing; Quad, quadriceps femoris; Sol, soleus; TSS, transcription start site.
(TIF)

S3 Fig Validation of transcription factors predicted by motif analysis. (A) qPCR expression analysis of transcription factors predicted from motif analyses in Quad and Sol (*n* = 5/group). Data are represented as means ± SEM. **p < 0.01, ***p < 0.001. (B) Histograms of NFAT tags within 1.5 kb of Sol-specific H3K27ac peak centers in Quad and Sol (left); quantification of NFAT tag densities at Sol-specific H3K27ac peaks in Quad and Sol; ***p < 0.001 (right). (C) Representative UCSC browser tracks of H3K27ac (top) and NFAT (bottom) ChIP-seq in Quad (yellow) and Sol (red) along a Sol-specific H3K27ac region with predicted NFAT motif (green) showing NFAT binding specifically in Sol. (D) ChIP qPCR validation of SIX1 binding in Quad (left) and Sol (right). Enrichment is plotted as percent of input using technical duplicate control IgG and SIX1 ChIPs. Numerical values for all panels are available in S12 Data. ChIP, chromatin immunoprecipitation; ChIP-seq, ChIP sequencing; H3K27ac, histone 3 lysine 27 acetylation; IgG, immunoglobulin G; NFAT, nuclear factor of activated T cells; qPCR, quantitative PCR; Quad, quadriceps femoris; SIX, sine oculis homeobox factor; Sol, soleus; UCSC, University of California, Santa Cruz.
(TIF)

S4 Fig PGC1α-modified epigenomic regions are hyperacetylated and enriched for MEF2 and ERR sites. (A) Wheel counts of mice during 4 weeks of voluntary exercise (*n* = 13). (B) Scatterplots of normalized H3K4me2 tag counts at genomic regions marked by H3K4me2 in pairwise comparisons of Sed control versus Ex quadriceps (left) and Wt versus mTg quadriceps (right). Correlation coefficient (r_s_) was calculated for each scatterplot (*n* = 2/group). (C) Comparison matrix listing the numbers of differential H3K4me2 peaks in Ex or mTg Quads compared with controls. (D) Histograms of H3K27ac tags within 6 kb of EMR peak centers (top) and quantification of H3K27ac tag densities at EMR peaks (bottom) in control mice and detrained mice that were removed from running wheels for 72 hours following their last bout of exercise; *n* = 2/group; ***p < 0.001. (E) Histograms showing distribution of H3K27ac tags within 6 kb of PMR peak centers in mTg and Ex mice and their respective controls; *n* = 2/group. (F) Quantification of H3K4me2 (left) and H3K27ac (right) tag densities at PMRs in Wt, mTg, Sed, and Ex Quads. *n* = 2/group, ***p < 0.001. (G) Relative gene expression of immune cell, vascular endothelial, satellite cell, and myogenic markers in quadriceps from control, Ex, and mTg mice (*n* = 5/group). Data are represented as means ± SEM. **p < 0.01, ***p < 0.001. (H) Top ontologies for genes annotated to hyperacetylated (top) or hypoacetylated (bottom) PMRs in mTg Quads. (I) Motifs enriched in H3K27ac peaks that are specifically hyperacetylated (left) and hypoacetylated (right) in mTg Quads. Numerical values for all panels are available in S12 Data. EMR, exercise-modified region; ERR, estrogen-related receptor; Ex, exercised; H3K4me2, histone 3 lysine 4 dimethylation; H3K27ac, histone 3 lysine 27 acetylation; MEF2, myocyte enhancer factor 2; mTg, muscle-specific *Pgc1a* transgenic; PGC1α, peroxisome proliferator–activated receptor gamma, coactivator-1 alpha; PMR, PGC1α-modified region; Quad, quadriceps femoris; Sed, sedentary; Wt, wild-type.
(TIF)

S5 Fig DNA footprints identified in Ex and mTg mice. (A) Activity scores for footprints in control versus Ex. Factors producing a significant activity score are shown in red, and motifs for the top 10 are shown below. (B) Activity scores for footprints in control versus mTg. Factors producing a significant activity score are shown in red, and motifs for the top 10 are shown below. Numerical values for all panels are available in S12 Data. Ex, exercised; mTg, muscle-specific *Pgc1a* transgenic.
(TIF)

S6 Fig Exercise or *Pgc1a* overexpression increases muscle oxidative fiber content. (A) Representative images of SDH staining in gastrocnemius of control, Ex, and mTg mice (*n* = 2/group). (B) Representative immunofluorescence staining images of four Myh isoforms in gastrocnemius from control, Ex, and mTg mice. Myh7 (type I) is blue, Myh2 (type IIa) is green, Myh1 (type IIx) is black, and Myh4 (type IIb) is red. Vertical bars next to the images show percentages of the different fiber types (*n* = 2/group). Ex, exercised; mTg, muscle-specific *Pgc1a* transgenic; *Myh*, myosin heavy chain; *Pgc1a*, peroxisome proliferator–activated receptor gamma, coactivator 1 alpha; SDH, succinate dehydrogenase.
(TIF)

S1 Table List of all significant transcription factors identified by IMAGE analysis in exercise reprogramming. Rows arranged in ascending order of *p*-value. IMAGE, integrated analysis of motif activity and gene expression.
(PDF)

S2 Table List of top 50 significant transcription factors identified by IMAGE analysis in PGC1α-induced reprogramming. Rows arranged in ascending order of *p*-value. IMAGE, integrated analysis of motif activity and gene expression.
(PDF)

S3 Table Read counts for all ChIP-seq samples. ChIP-seq, chromatin immunoprecipitation sequencing.
(PDF)

S4 Table List of ChIP qPCR primers. ChIP, chromatin immunoprecipitation; qPCR, quantitative PCR.
(PDF)

S5 Table List of qPCR primers. qPCR, quantitative PCR.
(PDF)

S1 Data Numerical values for all main figures. (XLSX)

S2 Data Numerical values for Fig 1C_Only H3K4me2 peaks. H3K4me2, histone 3 lysine 4 dimethylation.
(ZIP)

S3 Data Numerical values for Fig 1C_ H3K4me2 and H3K27ac peaks. H3K4me2, histone 3 lysine 4 dimethylation; H3K27ac, histone 3 lysine 27 acetylation.
(ZIP)

S4 Data Numerical values for Fig 1C_Only H3K27ac peaks. H3K27ac, histone 3 lysine 27 acetylation.
(ZIP)

S5 Data Numerical values for Fig 2E_EDL. EDL, extensor digitorum longus.
(ZIP)

S6 Data Numerical values for Fig 2E_Quad. Quad, quadriceps femoris.
(ZIP)

S7 Data Numerical values for Fig 2E_Sol. Sol, soleus.
(ZIP)

S8 Data Numerical values for Fig 2E_Dia. Dia, diaphragm.
(ZIP)

S9 Data Lists of super-enhancers. (XLSX)

S10 Data Numerical values for Fig 5B. (XLSX)

S11 Data Numerical values for Fig 6E. (XLSX)

S12 Data Numerical values for all supplemental figures. (XLSX)

## Abbreviations

Acadl: acyl-CoA dehydrogenase long chain
Acta1: actin alpha-1
AKT: protein kinase B
AMPK: AMP-kinase
ARID: AT-rich interaction domain
ATAC: assay for transposase-accessible chromatin
ATAC-seq: ATAC sequencing
ATF4: activating transcription factor 4
AUF1: AU rich element RNA binding protein 1
BACH: BTB domain and CNC homolog
Bdh1: 3-hydroxybutyrate dehydrogenase 1
BETA: Binding and Expression Target Analysis
BMP: bone morphogenetic protein
bZIP: basic leucine zipper domain factors
ChIP: chromatin immunoprecipitation
ChIP-seq: ChIP sequencing
CPEB1: cytoplasmic polyadenylation element binding protein 1
CTF: CCAAT box-binding transcription factor
Dia: diaphragm
E2F: E2F transcription factors
ECM: extracellular matrix
EDL: extensor digitorum longus
EMR: exercise-modified region
ERR: estrogen-related receptor
ETC: electron transport chain
ETS: E-twenty-six family of transcription factors
Ex: exercised
FA: fatty acid
FGFR: fibroblast growth factor receptor
FOXO4: forkhead box protein O4
GZF1: GDNF inducible zinc finger protein 1
H3K4: histone 3 lysine 4
H3K4me1: H3K4 monomethylation
H3K4me2: H3K4 dimethylation
H3K27: histone 3 lysine 27
H3K27ac: H3K27 acetylation
H3K64ac: histone 3 lysine 64 acetylation
H3K122ac: histone 3 lysine 122 acetylation
H4K16ac: histone 4 lysine 16 acetylation
HDAC: histone deacetylase
HIF: hypoxia inducible factor
HINT: Hmm-based identification of transcription factor footprints
HSF4: heat shock transcription factor 4
IMAGE: integrated analysis of motif activity and gene expression
Jun: jun proto-oncogene
KB: ketone body
MAFB: MAF bZIP transcription factor B
MAPK: mitogen-activated protein kinase
Mb: myoglobin
MED1: mediator complex subunit 1
MEF: myocyte enhancer factor
MRF: myogenic regulatory factor
mRNA-seq: mRNA sequencing
MSigDB: molecular signatures database
MT: myotube
mTg: muscle-specific *Pgc1a* transgenic
mTOR: mammalian target of rapamycin
Myh: myosin heavy chain
MyoD: myogenic differentiation 1
MYF5: myogenic factor 5
MyoG: myogenin
NFAT: nuclear factor of activated T cells
NFKB2: nuclear factor kappa B subunit 2
NFY: nuclear transcription factor Y
Ngdn: neuroguidin
NKX2: NK2 homeobox
NR: nuclear receptor
NRF: nuclear respiratory factor
NUR77: nuclear hormone receptor 77
PAX: paired box
PBX1: pre-B-cell leukemia homeobox
PDGF: platelet-derived growth factor
PDGFR: PDGF receptor
PGC1: peroxisome proliferator–activated receptor gamma, coactivator 1
PMR: PGC1α-modified region
PPAR: peroxisome proliferator–activated receptor
Pvalb: parvalbumin
qPCR: quantitative PCR
Quad: quadriceps femoris
RARβ: retinoic acid receptor beta
RUNX: runt-related transcription factor
RXR: retinoid X receptor
SE: super-enhancer
SF1: steroidogenic factor 1
SIX: sine oculis homeobox factor
Sol: soleus
SP1: specificity protein 1
STAT5A: signal transducer and activator of transcription 5A
TBX15: T-Box 15
TCA: citric acid cycle
TCF3: transcription factor 3
TEAD: TEA domain transcription factor 1
TF: transcription factor
TG: triacylglycerol
Tn5: transposase 5
TSS: transcription start site
Vegfa: vascular endothelial growth factor A
ZFP91: zinc finger protein 91
