# Supplementary figures and images for: Dynamic enhancers control skeletal muscle identity and reprogramming
Source: PLoS Biol. 2019 Oct 7;17(10):e3000467. doi: 10.1371/journal.pbio.3000467 (PMC6799888; doi:10.1371/journal.pbio.3000467)

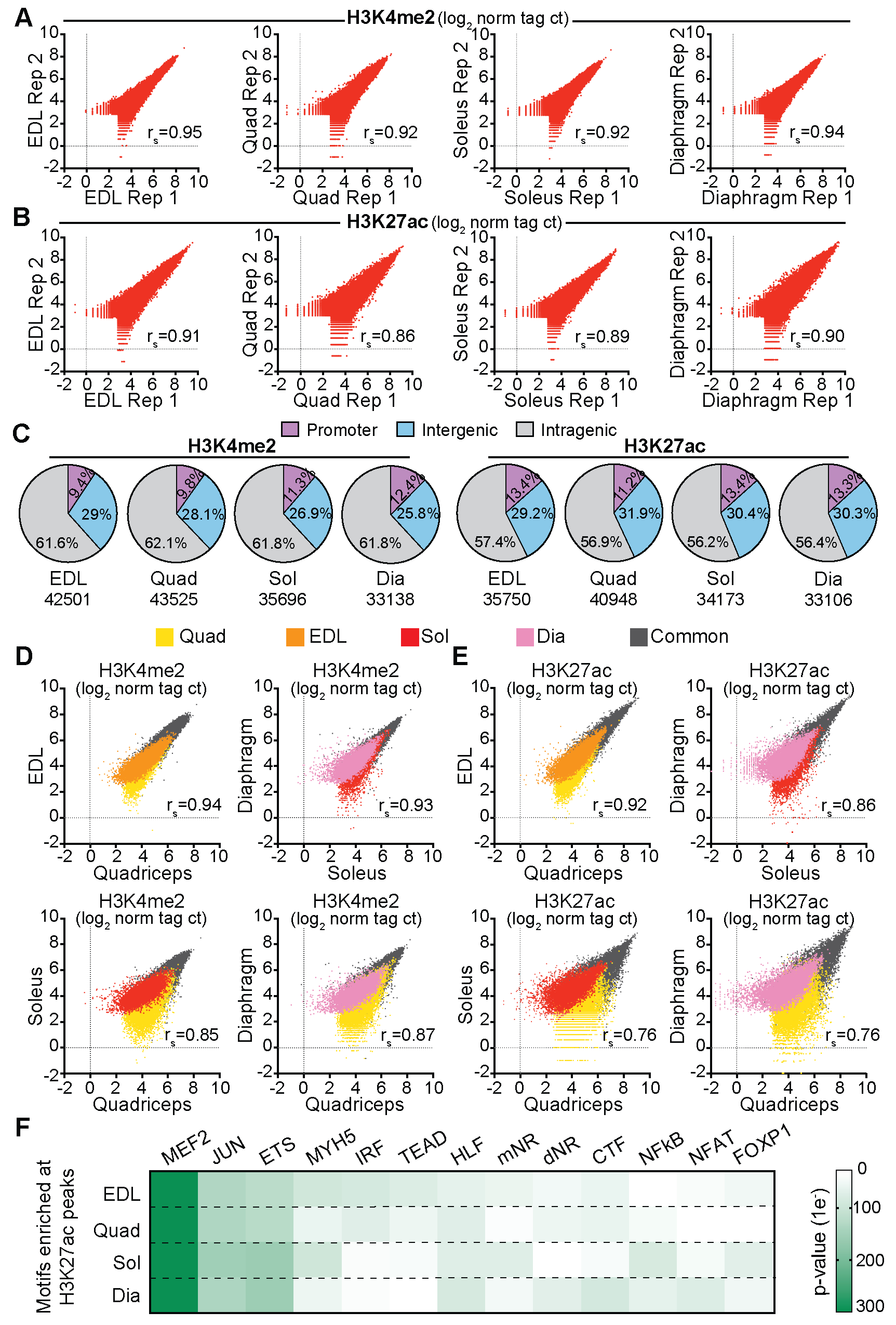

Supplement: S1 Fig — (A,B) Scatterplots of normalized H3K4me2 (A) and H3K27ac (B) tag counts for individual ChIP-seq replicates in the four muscle groups. Correlation coefficient (rs) was calculated for each scatterplot. (C) Genomic distributions of H3K4me2 (left) and H3K27ac (right) peaks across the four muscle groups in promoter, intragenic, and intergenic regions. (D,E) Scatterplots of normalized H3K4me2 (D) and H3K27ac (E) tag counts at genomic regions marked by H3K4me2 and H3K27ac tags in pairwise comparisons of four different muscle groups. Correlation coefficient (rs) was calculated for each scatterplot (n = 2/group). (F) Heatmap of motifs enriched in H3K27ac peaks for EDL, Quad, Sol, and Dia, with p-values represented in color. Numerical values for all panels are available in S12 Data. ChIP-seq, chromatin immunoprecipitation sequencing; Dia, diaphragm; EDL, extensor digitorum longus; H3K4me2, histone 3 lysine 4 dimethylation; H3K27ac, histone 3 lysine 27 acetylation; Quad, quadriceps femoris; Sol, soleus. (TIF) [file pbio.3000467.s001.tif]

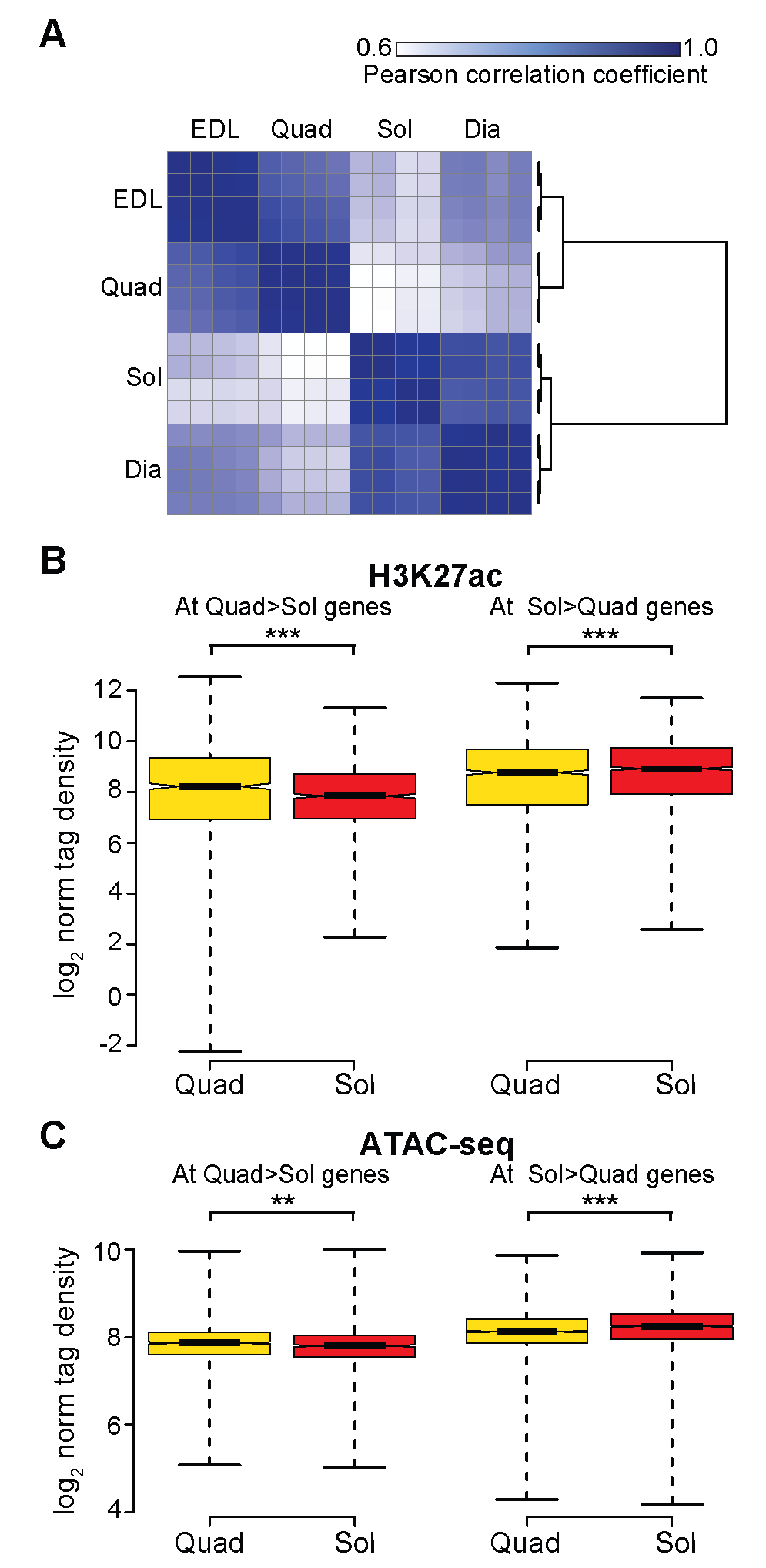

Supplement: S2 Fig — (A) Similarity matrix for Fpkm values from RNA-seq data for EDL, Quad, Dia, and Sol. Pearson correlation coefficient (r) is represented in color; n = 4/group. (B) H3K27ac tags within 50 kb around the TSS for genes differentially expressed (log2 fold change > 1.5) in Quad > Sol (left) or Sol > Quad (right); n = 2/group, ***p < 0.001. (C) ATAC-seq tags within 50 kb around the TSS for genes differentially expressed (log2 fold change > 1.5) in Quad > Sol (left) or Sol > Quad (right); n = 2/group, **p < 0.01, ***p < 0.001. Numerical values for all panels are available in S12 Data. ATAC-seq, assay for transposase-accessible chromatin sequencing; Dia, diaphragm; EDL, extensor digitorum longus; Fpkm, Fragments Per Kilobase of transcript per Million mapped reads; H3K27ac, histone 3 lysine 27 acetylation; RNA-seq, RNA sequencing; Quad, quadriceps femoris; Sol, soleus; TSS, transcription start site. (TIF) [file pbio.3000467.s002.tif]

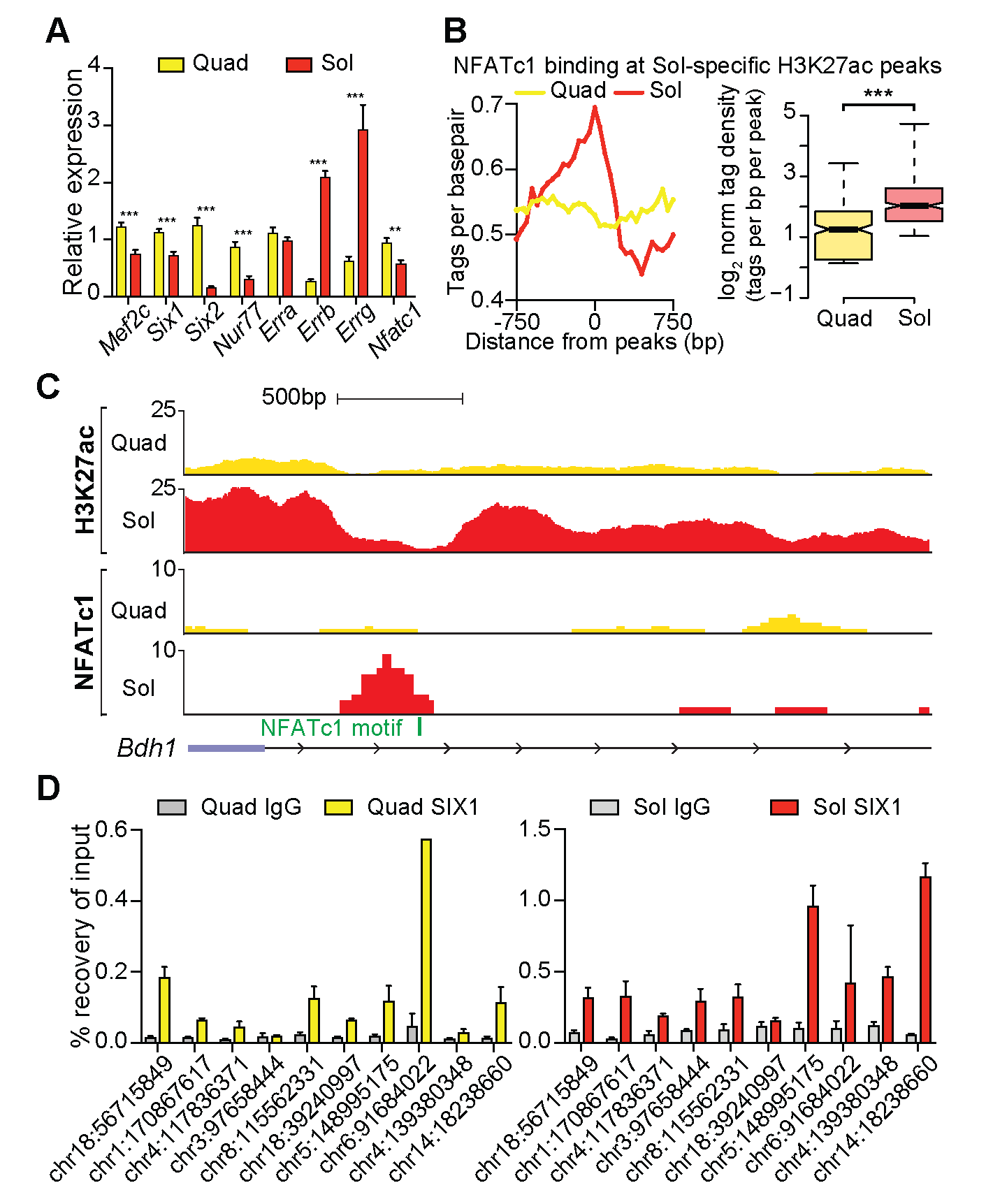

Supplement: S3 Fig — (A) qPCR expression analysis of transcription factors predicted from motif analyses in Quad and Sol (n = 5/group). Data are represented as means ± SEM. **p < 0.01, ***p < 0.001. (B) Histograms of NFAT tags within 1.5 kb of Sol-specific H3K27ac peak centers in Quad and Sol (left); quantification of NFAT tag densities at Sol-specific H3K27ac peaks in Quad and Sol; ***p < 0.001 (right). (C) Representative UCSC browser tracks of H3K27ac (top) and NFAT (bottom) ChIP-seq in Quad (yellow) and Sol (red) along a Sol-specific H3K27ac region with predicted NFAT motif (green) showing NFAT binding specifically in Sol. (D) ChIP qPCR validation of SIX1 binding in Quad (left) and Sol (right). Enrichment is plotted as percent of input using technical duplicate control IgG and SIX1 ChIPs. Numerical values for all panels are available in S12 Data. ChIP, chromatin immunoprecipitation; ChIP-seq, ChIP sequencing; H3K27ac, histone 3 lysine 27 acetylation; IgG, immunoglobulin G; NFAT, nuclear factor of activated T cells; qPCR, quantitative PCR; Quad, quadriceps femoris; SIX, sine oculis homeobox factor; Sol, soleus; UCSC, University of California, Santa Cruz. (TIF) [file pbio.3000467.s003.tif]

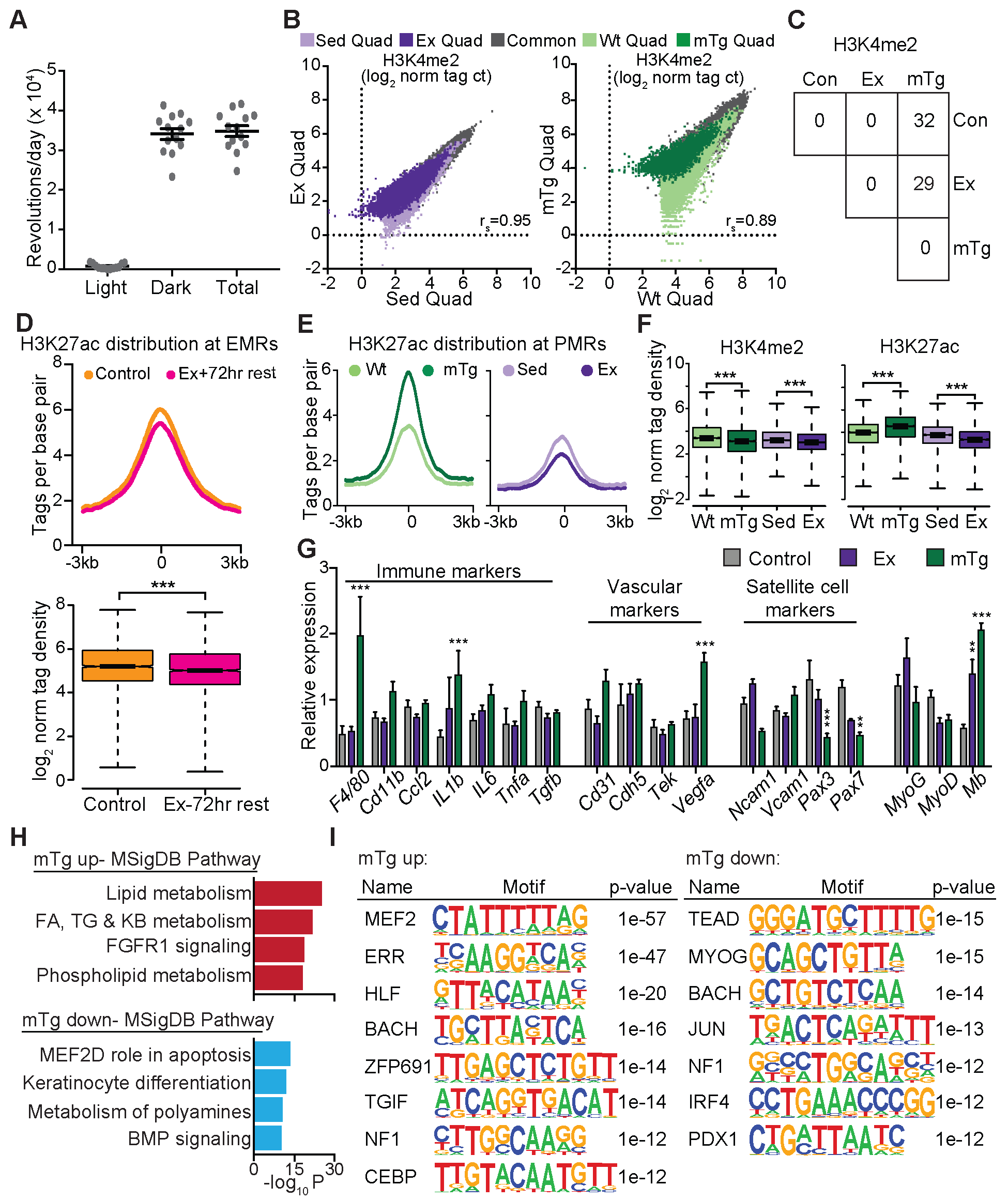

Supplement: S4 Fig — (A) Wheel counts of mice during 4 weeks of voluntary exercise (n = 13). (B) Scatterplots of normalized H3K4me2 tag counts at genomic regions marked by H3K4me2 in pairwise comparisons of Sed control versus Ex quadriceps (left) and Wt versus mTg quadriceps (right). Correlation coefficient (rs) was calculated for each scatterplot (n = 2/group). (C) Comparison matrix listing the numbers of differential H3K4me2 peaks in Ex or mTg Quads compared with controls. (D) Histograms of H3K27ac tags within 6 kb of EMR peak centers (top) and quantification of H3K27ac tag densities at EMR peaks (bottom) in control mice and detrained mice that were removed from running wheels for 72 hours following their last bout of exercise; n = 2/group; ***p < 0.001. (E) Histograms showing distribution of H3K27ac tags within 6 kb of PMR peak centers in mTg and Ex mice and their respective controls; n = 2/group. (F) Quantification of H3K4me2 (left) and H3K27ac (right) tag densities at PMRs in Wt, mTg, Sed, and Ex Quads. n = 2/group, ***p < 0.001. (G) Relative gene expression of immune cell, vascular endothelial, satellite cell, and myogenic markers in quadriceps from control, Ex, and mTg mice (n = 5/group). Data are represented as means ± SEM. **p < 0.01, ***p < 0.001. (H) Top ontologies for genes annotated to hyperacetylated (top) or hypoacetylated (bottom) PMRs in mTg Quads. (I) Motifs enriched in H3K27ac peaks that are specifically hyperacetylated (left) and hypoacetylated (right) in mTg Quads. Numerical values for all panels are available in S12 Data. EMR, exercise-modified region; ERR, estrogen-related receptor; Ex, exercised; H3K4me2, histone 3 lysine 4 dimethylation; H3K27ac, histone 3 lysine 27 acetylation; MEF2, myocyte enhancer factor 2; mTg, muscle-specific Pgc1a transgenic; PGC1α, peroxisome proliferator–activated receptor gamma, coactivator-1 alpha; PMR, PGC1α-modified region; Quad, quadriceps femoris; Sed, sedentary; Wt, wild-type. (TIF) [file pbio.3000467.s004.tif]

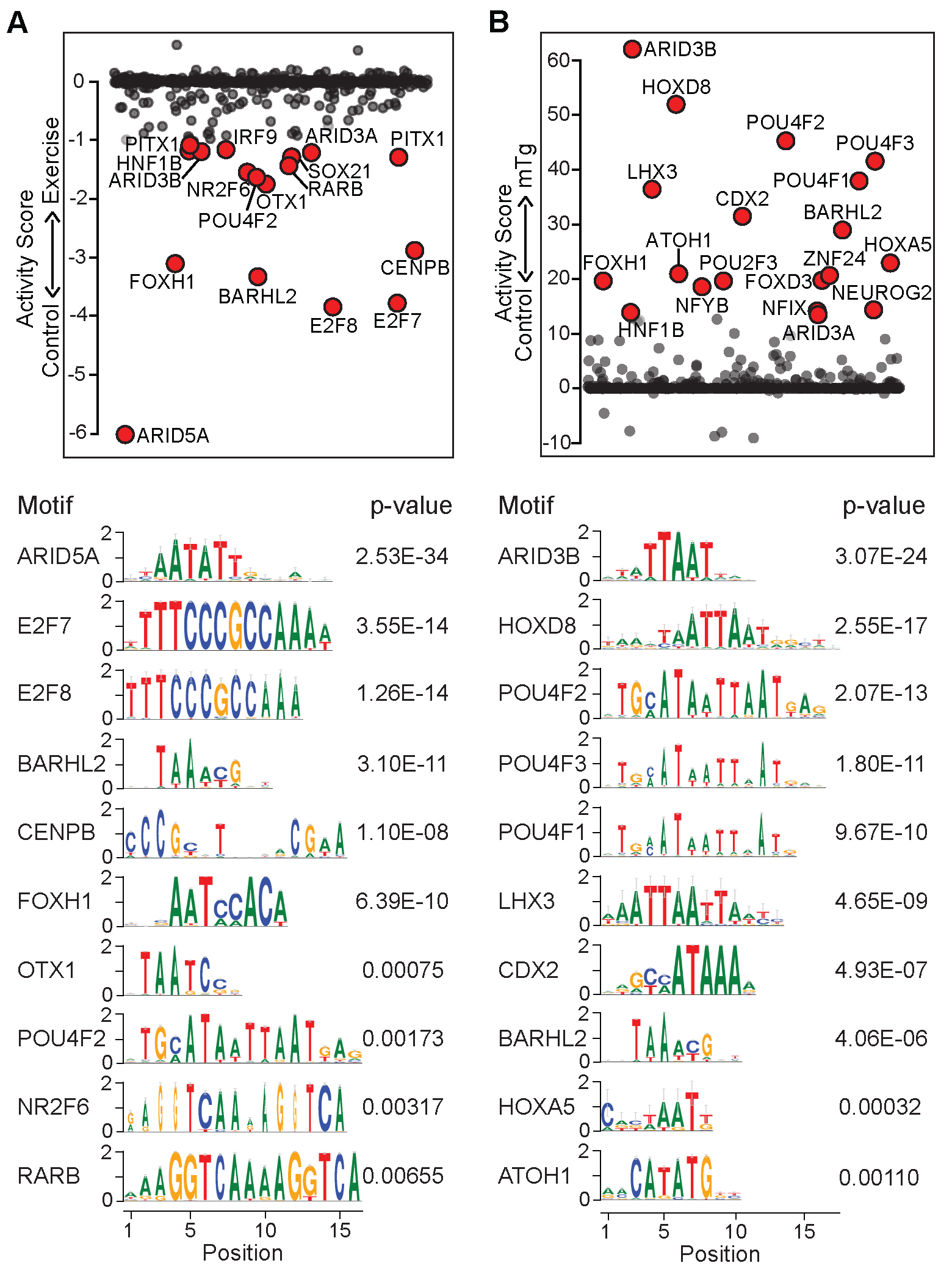

Supplement: S5 Fig — (A) Activity scores for footprints in control versus Ex. Factors producing a significant activity score are shown in red, and motifs for the top 10 are shown below. (B) Activity scores for footprints in control versus mTg. Factors producing a significant activity score are shown in red, and motifs for the top 10 are shown below. Numerical values for all panels are available in S12 Data. Ex, exercised; mTg, muscle-specific Pgc1a transgenic. (TIF) [file pbio.3000467.s005.tif]

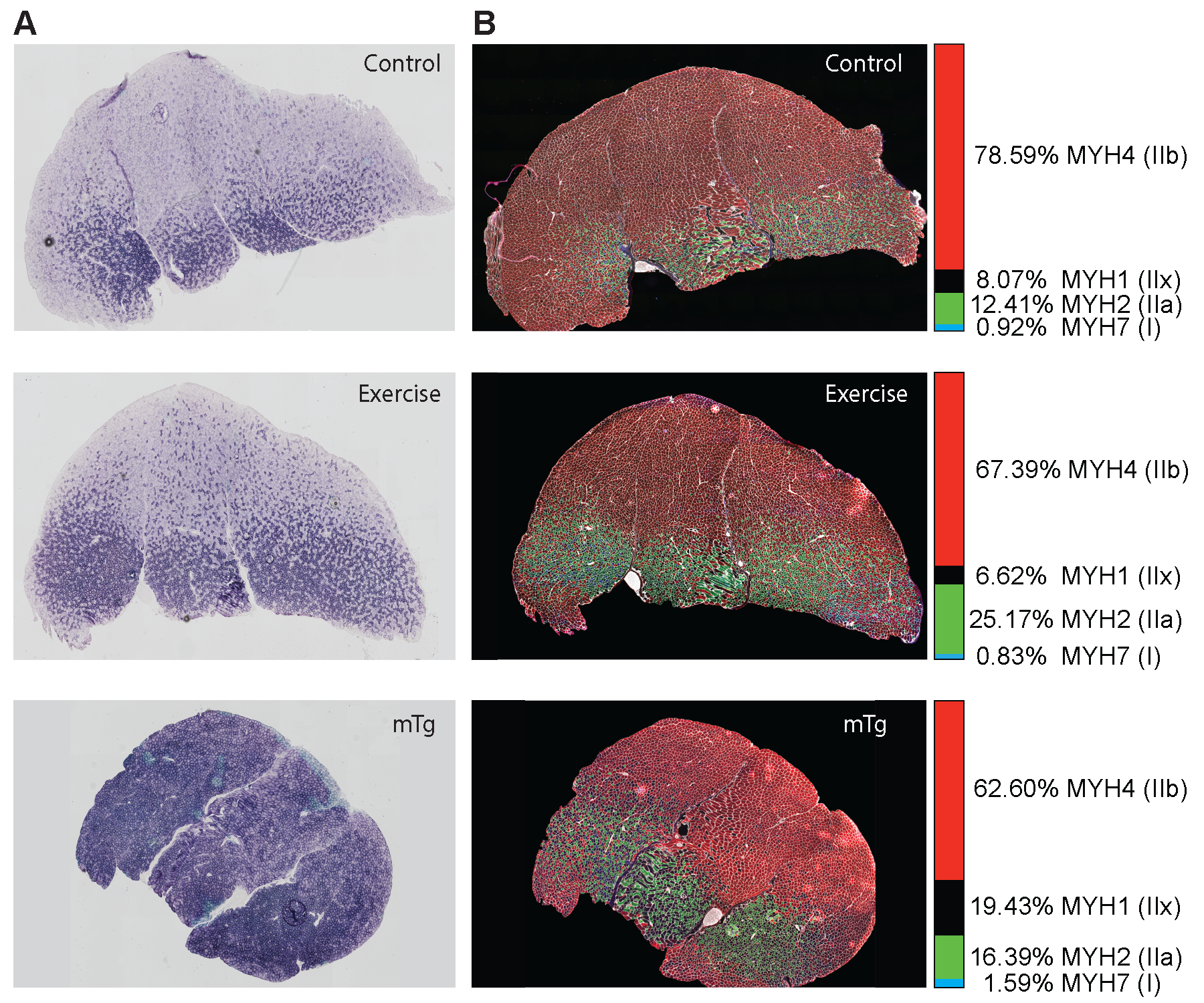

Supplement: S6 Fig — (A) Representative images of SDH staining in gastrocnemius of control, Ex, and mTg mice (n = 2/group). (B) Representative immunofluorescence staining images of four Myh isoforms in gastrocnemius from control, Ex, and mTg mice. Myh7 (type I) is blue, Myh2 (type IIa) is green, Myh1 (type IIx) is black, and Myh4 (type IIb) is red. Vertical bars next to the images show percentages of the different fiber types (n = 2/group). Ex, exercised; mTg, muscle-specific Pgc1a transgenic; Myh, myosin heavy chain; Pgc1a, peroxisome proliferator–activated receptor gamma, coactivator 1 alpha; SDH, succinate dehydrogenase. (TIF) [file pbio.3000467.s006.tif]
