# Supplementary material for: Dynamic enhancers control skeletal muscle identity and reprogramming
Source: PLoS Biol. 2019 Oct 7;17(10):e3000467. doi: 10.1371/journal.pbio.3000467 (PMC6799888; doi:10.1371/journal.pbio.3000467)
Supplement: S1 Table — Rows arranged in ascending order of p-value. IMAGE, integrated analysis of motif activity and gene expression. (PDF) [file pbio.3000467.s007.pdf]

S1 Table. List of all significant transcription factors identified by IMAGE analysis in exercise reprograming. (Rows arranged in ascending order of p-value.)

	Factor	Evidence	Activity_inSed	Activity_inEx	p-value	Pearsons	CausalTF
1	ESRRG	Direct	0.001226	0.009415	0.000023	0.936994	1
2	RXRA	Direct	0.032349	0.039294	0.000258	-0.813379	1
3	JUN	Direct	0.012241	0.017905	0.000778	-0.673597	1
4	NFKB2	Direct	0.044944	0.044095	0.001005	-0.517904	0
5	FOXO4	Direct	0.037628	0.039599	0.001019	-0.192316	2
6	MEF2A	Direct	0.085967	0.081144	0.001084	0.772731	0
7	NFYB	Direct	-0.002542	0.001666	0.001374	-0.926917	2
8	RXRG	Direct	0.029901	0.028413	0.001468	0.687594	0
9	ZFP91	ZifRC	0.032749	0.036491	0.001567	-0.093657	2
10	RXRG	Direct	0.028879	0.029217	0.001580	-0.110128	0
11	YBX3	Inferred	0.003568	0.001998	0.001984	0.157860	0
12	TEAD	Direct	0.030411	0.024867	0.002108	0.761156	2
13	CPEB1	Direct	0.001162	0.001207	0.002418	0.008779	0
14	STAT5A	Direct	0.020369	0.015298	0.002494	-0.829644	2
15	ATF4	Direct	0.000222	-0.003116	0.002990	0.567762	2
16	TBX15	Direct	0.043351	0.041871	0.003092	-0.377435	0
17	RXRA	Direct	0.034438	0.034282	0.003489	0.222462	0
18	GZF1	ZifRC	0.001339	-0.001024	0.004195	-0.865820	2
19	RXRA	Direct	0.002752	0.005662	0.004290	-0.787371	2
20	HSF4	Direct	-0.005625	-0.008901	0.004357	0.748361	2
21	ESRRB	Direct	-0.010043	-0.004626	0.004542	0.700247	2
22	YBX1	Direct	0.003568	0.001998	0.004544	0.620336	0
23	MBD2	Direct	0.014477	0.006401	0.005184	0.957802	0
24	RARG	Direct	-0.020541	-0.008586	0.005194	-0.808786	0
25	JUN	Direct	-0.005132	-0.002221	0.005302	-0.728326	0
26	HSF1	Direct	0.040311	0.036795	0.005874	0.498638	0
27	NFE2L1	Direct	-0.005177	-0.003519	0.006248	0.972995	0
28	E2F4	Direct	0.033352	0.033060	0.006359	0.274743	0
29	ARNT2	Direct	0.051222	0.048838	0.006781	0.106639	0
30	HBP1	Direct	0.009943	0.002436	0.008865	0.955809	0
31	MYF6	Direct	-0.011504	-0.008472	0.010080	-0.756211	0
32	RORA	Direct	0.019063	0.022970	0.010829	-0.686238	0
33	NFATC1	Direct	-0.006481	0.000754	0.013100	-0.575550	0
34	SOX4	Direct	0.008397	0.006868	0.013471	-0.582641	0
35	FOXO4	Direct	0.006683	0.005157	0.013515	0.165488	0
36	CBFB	Direct	-0.012534	-0.010536	0.013763	-0.904721	0
37	EPAS1	Direct	0.016785	0.015267	0.014466	-0.132116	0
38	SOX4	Direct	-0.002310	0.002099	0.016321	0.636383	0
39	ERG	Direct	-0.000099	-0.003915	0.017614	-0.669625	0
40	SIX2	Direct	-0.007117	-0.007265	0.020405	0.291730	0
41	HOXC11	Direct	0.010934	0.006901	0.020600	0.771530	0
42	MXI1	Direct	0.004303	0.001778	0.021010	0.955262	0
43	FOXO6	Direct	0.029145	0.031248	0.021414	0.191625	0
44	NR3C1	Direct	0.034980	0.034152	0.028322	0.226645	0
45	MNT	Direct	-0.010031	-0.011292	0.029993	0.085313	0
46	NR3C1	Direct	-0.010509	-0.012943	0.031238	0.414702	0
47	E2F4	Direct	-0.015990	-0.014068	0.039102	-0.527873	0
48	LYL1	Indirect	-0.003008	-0.005354	0.040162	-0.471484	0
49	PRKRIR	Direct	-0.011503	-0.009443	0.043112	-0.722880	0
50	HES6	Inferred	-0.026655	-0.022916	0.049140	0.713352	0

1= high confidence, 2= medium confidence, and 0=low confidence, hits for causal transcription factor.
