# Supplementary material for: Dynamic enhancers control skeletal muscle identity and reprogramming
Source: PLoS Biol. 2019 Oct 7;17(10):e3000467. doi: 10.1371/journal.pbio.3000467 (PMC6799888; doi:10.1371/journal.pbio.3000467)
Supplement: S2 Table — Rows arranged in ascending order of p-value. IMAGE, integrated analysis of motif activity and gene expression. (PDF) [file pbio.3000467.s008.pdf]

S2 Table. List of top 50 significant transcription factors identified by IMAGE analysis in PGC1a-induced reprogramming. (Rows arranged in ascending order of p-value.)

	Factor	Evidence	Activity_inWt	Activity_inmTg	p-value	Pearsons	CausalTF
1	ERRB	Direct	0.011855	0.028410	0.000000	0.995243	1
2	ERRA	Direct	-0.006167	-0.000977	0.000001	0.987824	1
3	ERRA	Direct	-0.001879	0.018145	0.000002	0.994505	1
4	NR4A1	Direct	-0.000758	0.002924	0.000005	-0.988116	1
5	ERRA	Direct	0.008213	0.012049	0.000006	0.968895	1
6	HOXB7	Direct	0.000593	0.029049	0.000037	0.917550	1
7	CPEB1	Direct	0.011292	0.014062	0.000071	-0.970365	0
8	ERRG	Direct	0.010888	0.027910	0.000115	0.580828	1
9	ERRA	Direct	0.004878	0.005189	0.000123	0.339694	0
10	SREBF1	Direct	0.013010	0.014185	0.000129	-0.826646	0
11	GZF1	ZifRC	0.005885	0.008132	0.000131	0.846568	1
12	RXRA	Direct	0.013480	0.021275	0.000148	-0.866814	0
13	SCX	Inferred	0.016561	0.002168	0.000176	0.918812	1
14	HOXC10	Direct	0.012543	0.016959	0.000179	-0.996328	0
15	RORA	Direct	0.014929	0.013631	0.000220	0.892558	1
16	CBFB	Direct	0.011302	0.012197	0.000247	-0.631139	0
17	NR1D1	Direct	-0.002993	0.002399	0.000352	-0.895112	1
18	YBX3	Inferred	-0.003281	-0.004372	0.000355	0.929864	0
19	SREBF1	Direct	0.003720	-0.000446	0.000357	0.880117	0
20	EPAS1	Direct	0.004394	0.002204	0.000394	-0.855816	0
21	PRDM16	Indirect	0.024891	0.028142	0.000397	0.675685	1
22	KLF13	Direct	0.014556	0.004047	0.000456	0.420846	1
23	MEF2D	Direct	0.016464	0.015813	0.000456	0.590686	0
24	FIZ1	ZifRC	0.001309	0.015676	0.000484	-0.895309	1
25	RORC	Direct	-0.005124	-0.001870	0.000515	0.997044	1
26	MAZ	Direct	-0.006179	-0.009788	0.000603	0.806545	1
27	STAT5B	Direct	-0.001462	-0.002725	0.000641	0.802747	0
28	SMAD3	Direct	0.002394	0.007839	0.000742	-0.720531	1
29	NR1D1	Direct	0.003940	0.009193	0.000742	-0.919652	0
30	RXRG	Direct	0.010301	0.010222	0.000764	-0.106066	0
31	BHLHE40	Direct	0.001500	0.000340	0.000908	0.828016	0
32	RXRA	Direct	-0.002953	0.001552	0.000908	-0.999591	1
33	TEAD1	Direct	0.003138	0.000266	0.000950	0.724083	1
34	TEAD1	Direct	0.006746	0.012400	0.000957	-0.706400	1
35	FOXJ2	Direct	0.016193	0.013109	0.000987	0.927055	0
36	HMG20B	Indirect	0.006636	0.010750	0.000991	0.895972	0
37	MEIS1	Direct	0.008020	0.015880	0.001011	-0.863478	2
38	KLF2	Inferred	0.014036	-0.008113	0.001032	0.543753	2
39	KLF4	Direct	0.014036	-0.008113	0.001067	0.875714	2
40	XBP1	Direct	0.001048	-0.002795	0.001184	0.837698	2
41	THRA	Direct	0.015662	0.017626	0.001241	-0.896301	0
42	FOXO4	Direct	0.005048	-0.000856	0.001353	0.524323	2
43	SOX7	Direct	0.015311	0.013043	0.001380	-0.741460	0
44	RXRG	Direct	0.009877	0.009659	0.001427	-0.033696	0
45	MXI1	Direct	0.019188	0.021708	0.001476	-0.913181	0
46	PRDM4	Direct	0.010601	0.011775	0.001503	-0.732325	0
47	MYF6	Direct	-0.003675	-0.006026	0.001521	0.885123	0
48	NFYB	Direct	0.010064	0.014836	0.001565	0.769750	2
49	TCF15	Direct	0.000861	-0.009745	0.001572	-0.700350	2
50	RXRA	Direct	-0.001389	0.001610	0.001574	-0.959115	0

1= high confidence, 2= medium confidence, and 0=low confidence, hits for causal transcription factor.
