# Supplementary material for: Dynamic enhancers control skeletal muscle identity and reprogramming
Source: PLoS Biol. 2019 Oct 7;17(10):e3000467. doi: 10.1371/journal.pbio.3000467 (PMC6799888; doi:10.1371/journal.pbio.3000467)
Supplement: S3 Table — ChIP-seq, chromatin immunoprecipitation sequencing. (PDF) [file pbio.3000467.s009.pdf]

**Supplemental Table S3**: Read counts for all ChIP-seq samples.

| Sample name | # of reads | # aligned reads | # unique reads |
|---|---|---|---|
| Dia-H3K27Ac1 | 27,228,932 | 24,410,500 (89.65%) | 21,478,782 |
| Dia-H3K27Ac2 | 23,013,847 | 20,861,970 (90.65%) | 18,165,700 |
| Dia-H3K4me2-1 | 21,382,324 | 18,637,118 (87.16%) | 17,246,922 |
| Dia-H3K4me2-2 | 22,607,319 | 19,604,794 (86.72%) | 19,189,217 |
| EDL-H3K27Ac1 | 23,957,367 | 21,798,545 (90.99%) | 19,824,832 |
| EDL-H3K27Ac2 | 26,196,222 | 23,833,611 (90.98%) | 21,602,302 |
| EDL-H3K4me2-1 | 25,921,784 | 22,513,200 (86.85%) | 20,971,524 |
| EDL-H3K4me2-2 | 24,244,906 | 21,045,468 (86.80%) | 19,496,114 |
| Quad-H3K27Ac1 | 30,149,431 | 27,470,836 (91.12%) | 24,195,147 |
| Quad-H3K27Ac2 | 22,622,781 | 20,202,090 (89.30%) | 15,271,950 |
| Quad-H3K4me2-1 | 29,135,549 | 25,537,375 (87.65%) | 22,731,207 |
| Quad-H3K4me2-2 | 25,213,895 | 22,353,350 (88.65%) | 19,660,934 |
| Sol-H3K27Ac1 | 25,724,160 | 21,883,531 (85.07%) | 20,277,170 |
| Sol-H3K27Ac2 | 25,009,280 | 21,707,399 (86.80%) | 19,783,028 |
| Sol-H3K4me2-1 | 21,893,402 | 17,979,294 (82.12%) | 16,849,204 |
| Sol-H3K4me2-2 | 30,773,295 | 25,579,368 (83.12%) | 22,382,835 |
| Dia-input | 30,259,239 | 24,223,214 (80.05%) | 19,581,205 |
| EDL-input | 27,930,681 | 21,866,076 (78.29%) | 19,857,682 |
| Quad1-Input | 22,721,692 | 18,127,990 (79.78%) | 12,578,289 |
| Quad2-Input | 23,327,883 | 18,658,213 (79.98%) | 15,825,420 |
| Sol-input | 27,560,985 | 21,271,015 (77.18%) | 18,901,362 |
| Sed-H3K27Ac1 | 29,187,911 | 25,865,792 (88.62%) | 23,663,625 |
| Sed-H3K27Ac2 | 31,260,255 | 27,767,394 (88.83%) | 20,735,446 |
| Ex-H3K27Ac1 | 31,521,546 | 28,265,381 (89.67%) | 18,546,989 |
| Ex-H3K27Ac2 | 28,173,841 | 18,350,387 (86.85%) | 17,145,917 |
| SedAc-input | 28,043,774 | 21,973,381 (78.35%) | 20,918,250 |
| ExAc-input | 32,272,408 | 24,882,471 (77.10%) | 22,883,142 |
| Sed-H3K4me2-1 | 28,433,466 | 23,496,908 (82.64%) | 20,118,788 |
| Sed-H3K4me2-2 | 28,408,046 | 24,021,928 (84.56%) | 19,560,570 |
| Ex-H3K4me2-1 | 30,466,545 | 25,179,228 (82.65%) | 21,140,891 |
| Ex-H3K4me2-2 | 31,017,911 | 25,843,450 (83.32%) | 21,639,809 |
| SedMe2_input | 28,773,134 | 21,956,118 (76.31%) | 19,307,672 |
| ExMe2_input | 25,368,203 | 19,406,436 (76.50%) | 17,349,120 |
| Wt-H3K27Ac1 | 22,857,995 | 18,620,546 (81.46%) | 14,942,618 |
| Wt-H3K27Ac2 | 17,996,149 | 15,739,534 (87.46%) | 14,068,601 |
| Wt-H3K4me2-1 | 18,389,196 | 14,524,361 (78.98%) | 13,722,774 |
| Wt-H3K4me2-2 | 18,431,343 | 14,511,203 (78.73%) | 13,259,102 |
| mTg-H3K27Ac1 | 19,006,769 | 16,618,221 (87.43%) | 11,658,746 |
| mTg-H3K27Ac2 | 16,910,538 | 14,849,485 (87.81%) | 12,685,576 |
| mTg-H3K4me2-1 | 16,633,836 | 11,341,331 (68.18%) | 6,405,517 |
| mTg-H3K4me2-2 | 20,393,257 | 15,322,435 (75.13%) | 14,127,860 |
| Wt-input | 17,837,791 | 13,196,696 (73.98%) | 7,721,156 |
| mTg-input | 20,619,561 | 15,286,765 (74.14%) | 8,826,665 |
