# Supplementary material for: Dynamic enhancers control skeletal muscle identity and reprogramming
Source: PLoS Biol. 2019 Oct 7;17(10):e3000467. doi: 10.1371/journal.pbio.3000467 (PMC6799888; doi:10.1371/journal.pbio.3000467)
Supplement: S4 Table — ChIP, chromatin immunoprecipitation; qPCR, quantitative PCR. (PDF) [file pbio.3000467.s010.pdf]

S4 Table. List of ChIP-qPCR primers.

**Location (*gene name*)**	**Forward 5'-3'**	**Reverse 5'-3'**
chr18:56,715,849-56,716,049 (*Lmbd1*)	GGACTGCCGTGATCAAAGA	AGAGGCTCCTGAATGTTTGTAG
chr1:170,867,617-170,867,836 (*Atf6*)	AAACAGACACCTCTCCCTCA	CGTGTCAGCGTTCAGCTTAT
chr4:117,836,371-117,836,590 (*Slc6a9*)	CACGTGGTGGCTGATGTAA	CCACTTCCTCGAAACGAGAC
chr3:97,658,444-97,658,645 (*Prkab2*)	CCCGCCTGGTGTCATTC	CGCTCGCTGGTAGTGTTT
chr8:115,562,331-115,562,550 (*4930488N15Rik*)	CAACAAAGCCAGATCAGAAACC	AGCAACAGAGGACAAGGATTAG
chr18:39,240,997-39,241,216 (*Arhgap26*)	GACTCTCCTCCCTGTAATGTTATG	CTGTTCCATGAACCGCATTTAG
chr5:148,995,175-148,995,394 (*5930430L01Rik*)	GATGGTTTCTCCTCTTTGAATAAGC	CAGGCAATCACGTTGCAC
chr6:91,684,022-91,684,241 (*Slc6a6*)	GTCAGGGAGAAGCCGCTTATAAAT	GAGCTCGGCAGCAACCA
chr4:139,380,348-139,380,567 (*Ubr4*)	GGAACCATATTGCCGCTTATTG	CTAGGAGCAATGCCCTCTG
chr14:18,238,660-18,238,859 (*Nr1d2*)	CACGCGAGATGGACGCA	ATGGAGCTGAACGCAGGTAAA
chr6:134,512,826-134,513,045 (*Lrp6*)	GAAGTTTCCTAGTAAGCTGGTATTT	GACAGCTCATCTGCTGTTTATG
chr5:130,198,141-130,198,341 (*Rabgef1*)	ATAGGAGCTGTGGTTTAGGGA	AGGCCGGTCTTAGGTAACTATC
chr13:101,233,524-101,233,776 (*5930438M14Rik*)	GCTACTGCTGAGCCAACCAT	GCTACTGCTGAGCCAACCAT
chr6:53,287,157-53,287,366 (*Creb5*)	GTTCAGGAATCCCACGTGAC	CACGTTGGACTGAGCAAAGA
chr15:98,831,368-98,831,577 (*Prkag1*)	CCAGTTCCTCAGCTTGGAAAG	TCTGACGTAGGCGGAAGT
chr7:16,313,921-16,314,130 (*Bbc3*)	AGGAATGGATCTGCTGGATG	GGGCTCCGAGTAGCTTTC
chr11:67,104,787-67,105,061 (*Myh3*)	GACTCGATGGTGATGAGACG	CCTTGGGCAAGTTACCATGT
chr4:95,052,121-95,052,320 (*Jund*)	AGCCTGAGCTCAACACTTATC	AGAACGACGCAAGCCAAT
