# Supplementary material for: Dynamic enhancers control skeletal muscle identity and reprogramming
Source: PLoS Biol. 2019 Oct 7;17(10):e3000467. doi: 10.1371/journal.pbio.3000467 (PMC6799888; doi:10.1371/journal.pbio.3000467)
Supplement: S5 Table — qPCR, quantitative PCR. (PDF) [file pbio.3000467.s011.pdf]

S5 Table. List of qPCR primers.

Gene	Forward 5'-3'	Reverse 5'-3'
36b4	AGATGCAGCAGATCCGCA	GTTCTTGCCCATCAGCACC
Ccl2	CTTCTGGGCCTGCTGTTCA	CCAGCCTACTCATTGGGATCA
Cd11b	ATGGACGCTGATGGCAATACC	TCCCCATTCACGTCTCCCA
Cd31	CACCCATCACTTACCACCTTATG	TGTCTCTGGTGGGCTTATCT
Cdh5	CTTCAACGCTACCAAGCAAAC	GATGAGTTACAGCCTCCCTTTAG
Erra	TGCTCAGCTCTCTACCCAAAC	GGACAGCTGTACTCGATGCTC
Errb	CCGGCCACCAATGAATGT	ATCCAGCCGTCGCTTGTACT
Errg	ATGCCCAAGAGACTGTGCTT	CTTCTTTCAGCATGCCCACT
F4/80	TTGTACGTGCAACTCAGGACT	GATCCCAGAGTGTTGATGCAA
Il1b	CAGGATGAGGACATGAGCACC	CATGAGTCACAGAGGATGGGC
IL6	TCTATACCACTTCACAAGTCGGA	GAATTGCCATTGCACAACTCTTT
Mb	AGCTGGTGCTGAATGTCTGG	AACAGACCGATGAGGACTTCC
Mef2c	CAAATCTCTCCCTGCCTTCTAC	GTGGTGTGTTGTGGGTATCT
Myh1	GAGGGACAGTTCATCGATAGCAA	GGGCCAACTTGTCATCTCTCAT
Myh2	AGGCGGCTGAGGAGCACGTA	GCGGCACAAGCAGCGTTGG
Myh4	CACCTGGACGATGCTCTCAGA	GCTCTTGCTCGGCCACTCT
Myh7	CTCAAGCTGCTCAGCAATCTATTT	GGAGCGCAAGTTTGTCATAAGT
Myod	TCCGCTACATCGAAGGTCTG	GTCCAGGTGCGTAGAAGGC
Myog	GTGGGCATGTAAGGTGTGTAA	CGAAGGCCTCATTCACTTTCT
Ncam1	GCGAACTAAGGATCTCATCTGG	GGACTCTCCAACGCTGATTT
Nfatc1	TTCCGCAACCAGAGGATAAC	GGCAGGAAGGTACGTGAAA
Nur77	CTGTCACCCATGTGCCTTTA	TGAGGAGGTACGTCAGTCTTAG
Pax3	AACCCACTACCCAGACATTTAC	CCAGCTTGTTTCCTCCATCT
Pax7	CGTAAGCAGGCAGGAGCTAA	ACTGTGCTGCCTCCATCTTG
Six1	CCAGGTCAGCAACTGGTTTA	GCTTGTTGGAGGAGGAGTTATT
Six2	GTGAACGGAGGTAGACATTCTG	GCCTTTCTTGCTCATTTGTTAGT
Tek	TTTGCCCTCCTGGGTTTATG	CTTCTGGTCCACTACACCTTTC
Tgfb	CTCCCGTGGCTTCTAGTG	GCCTTAGTTTGGACAGGATCTG
Tnfa	ACTCCCAGGTTCTCTTCAAGG	GTGGGTGAGGAGCACGTAGT
Vcam1	GAGGGAGACACCGTCATTATC	CGAGCCATCCACAGACTTTA
Vegfa	CACTTCCAGAAACACGACAAAC	TGGAACCGGCATCTTTATCTC
